# Different pro-angiogenic potential of γ-irradiated PBMC-derived secretome and its subfractions

**DOI:** 10.1038/s41598-018-36928-6

**Published:** 2018-12-20

**Authors:** Tanja Wagner, Denise Traxler, Elisabeth Simader, Lucian Beer, Marie-Sophie Narzt, Florian Gruber, Sibylle Madlener, Maria Laggner, Michael Erb, Vera Vorstandlechner, Alfred Gugerell, Christine Radtke, Massimiliano Gnecchi, Anja Peterbauer, Maria Gschwandtner, Erwin Tschachler, Claudia Keibl, Paul Slezak, Hendrik J. Ankersmit, Michael Mildner

**Affiliations:** 1 Division of Thoracic Surgery, Medical University of Vienna, Vienna, Austria; 2 Department of Cardiology, Department of Internal Medicine II, Medical University of Vienna, Vienna, Austria; 3 Department of Biomedical Imaging and Image-guided Therapy, Medical University of Vienna, Vienna, Austria; 4 Research Division of Biology and Pathobiology of the Skin, Department of Dermatology, Medical University of Vienna, Vienna, Austria; 5 Molecular Neuro-Oncology Research Unit, Department of Pediatrics and Adolescent Medicine and Institute of Neurology, Medical University of Vienna, Vienna, Austria; 6 Synlab, Birsfelden, Switzerland; 7 Clinical Division of Plastic and Reconstructive Surgery, Department of Surgery, Medical University of Vienna, Vienna, Austria; 8 Department of Molecular Medicine, Unit of Cardiology, University of Pavia, Pavia, Italy; 9 Coronary Care Unit, Laboratory of Experimental Cardiology for Cell and Molecular Therapy, Fondazione IRCCS Policlinico San Matteo Foundation, Pavia, Italy; 10 Department of Medicine, University of Cape Town, Cape Town, South Africa; 11 Red Cross Blood Transfusion Service of Upper Austria, Linz, Austria; 12 Ludwig Boltzmann Institute for Experimental and Clinical Traumatology, Vienna, Austria; 13 FFG Project 852748 “APOSEC”, Medical University of Vienna, Vienna, Austria; 14 Christian Doppler Laboratory for Cardiac and Thoracic Diagnosis and Regeneration, Vienna, Austria

## Abstract

Secretomes from various cell sources exert strong regenerative activities on numerous organs, including the skin. Although secretomes consist of many diverse components, a growing body of evidence suggests that small extracellular vesicles (EVs) account for their regenerative capacity. We previously demonstrated that the secretome of γ-irradiated peripheral blood mononuclear cells (PBMCs) exhibits wound healing capacity. Therefore, we sought to dissect the molecular composition of EVs present in the secretome and compared wound healing-related activities of these EVs to other subfractions of the secretome and the fully supplemented secretome (MNC^aposec^). Compared to EVs derived from non-irradiated PBMCs, γ-irradiation significantly increased the size and number and changed the composition of released EVs. Detailed characterization of the molecular components of EVs, *i.e*. miRNA, proteins, and lipids, derived from irradiated PBMCs revealed a strong association with regenerative processes. Reporter gene assays and aortic ring sprouting assays revealed diminished activity of the subfractions compared to MNC^aposec^. In addition, we showed that MNC^aposec^ accelerated wound closure in a diabetic mouse model. Taken together, our results suggest that secretome-based wound healing represents a promising new therapeutic avenue, and strongly recommend using the complete secretome instead of purified subfractions, such as EVs, to exploit its full regenerative capacity.

## Introduction

In the past decade, many organs have demonstrated morphological plasticity and the capacity for healing instead of being post-mitotic terminally differentiated in preclinical and clinical trials of regenerative medicine utilizing stem cells^1–4^. Transplantation of adult stem cells has been proposed and tested to repair different kinds of tissue damage. The rationale for such an approach was derived from studies demonstrating the capacity of bone marrow-derived cells to transdifferentiate into various cells types, including neurons, cardiomyocytes, hepatocytes, bone and skin cells, just to name some^5,6^. Despite very promising pre-clinical results, meticulously performed first clinical trials did not fully meet the expectations and the use of adult stem cell therapy for organ regeneration has not entered the routine clinical practice^7^. More recently, cell transplantation has been shown to elicit tumor formation, graft versus host disease, unwanted immune responses, thrombosis, and infections^8,9^. The main reasons for what so far can be considered a failure are poor cell engraftment and low, if any, capacity to truly transdifferentiate into mature cells. Regulatory restrictions and high costs further complicate the whole scenario. Despite all that, we have learned a lot from these first experiences. The key finding was that adult stem cells exert beneficial paracrine actions, mediated by soluble factors produced and released by the transplanted cells, rather than through transdifferentiation^10–14^. The first clear demonstration of the paracrine hypothesis came from two studies in which the administration of conditioned medium from mesenchymal stem cells was able to repair cardiac damage^10,15^.

Few years later, in 2008, Timmers *et al*. demonstrated that fractionated conditioned medium products of mesenchymal stem cells and containing only soluble factors smaller that 100–200 nm in size provides cardio protection in an ischemia reperfusion (I/R) injury model^16^. The same group later demonstrated that extracellular vesicles (EVs) are responsible for a reduction in I/R injury in a Langendorff Model^17^. Recently, small EVs derived from stem cells or platelet-rich plasma were shown to significantly contribute to wound healing processes in different rodent models^18,19^.

The literature in regenerative medicine has recently undergone an oblique twist. The “stem cell-centric vision” was silently exchanged for a “stem cell-derived EV-centric vision”. As the availability and accessibility of stem cells of different origins are limited and it is time-consuming and extremely expensive to produce them under Good Manufacturing Practice (GMP) conditions^20^, we focused on peripheral blood mononuclear cells (PBMCs) as a source for secretome production. These cells can be obtained easily and are a current waste product in all transfusion units worldwide. In the past, we have shown that the secretome of γ-irradiated PBMCs (MNC^aposec^) has regenerative capacity in several preclinical settings. MNC^aposec^ abrogates hypoxia-induced cell damage in acute myocardial infarction and chronic ischemia, attenuates stroke and spinal injury, resolves myocarditis, and augments wound healing^21–27^. Multiple modes of action have been described, such as the induction of cytoprotection, inhibition of platelet aggregation, induction of vasodilation, endothelial cell proliferation, migration of epithelial and mesenchymal cells, and immune modulation and inhibition of bacterial growth^28^. More recently, we demonstrated the safety and tolerability of autologous MNC^aposec^ produced under GMP conditions in human full-thickness skin wounds^29^.

Diabetic wounds and impaired wound healing represent clinical challenges with an unmet need^30,31^. The process of wound healing involves well-orchestrated biological events, including macrophage invasion, reepithelization, fibroblast migration and proliferation, extracellular matrix deposition, and neo-angiogenesis^32–34^. However, in diabetic wounds, the healing process is impaired, which can lead to chronic non-healing wounds, such as diabetic foot ulcers, and, in the worst case, requires limb amputation^32^. Similar to myocardial infarction, the regenerative medicine literature on diabetic wound repair has undergone a change in paradigm. The “mesenchymal stem cell -based therapies” are currently superseded by “EV-based therapies” in experimental wound models^19,35–40^.

Two of our previous publications^27,41^ set the stage for the present study. We have conclusively shown that MNC^aposec^ is superior to the secretome of non-irradiated PBMCs in regards to the healing capacity in a porcine model of acute burn wounds, and that PBMCs release a plethora of proteins, lipids, and EVs into the culture medium, all with different biological activities^41^.

Therefore, the aims of the current study were: 1) to investigate whether γ-irradiation changes the miRNA, protein, and lipid content of EVs released by PBMCs; 2) to test if subfractions of MNC^aposec^ have better wound healing properties *in vitro* than the whole secretome; 3) to verify whether MNC^aposec^ exerts its regenerative activity in an animal diabetic wound model.

## Results

### PBMCs exhibit an altered EV secretion profile in response to ionizing radiation

**Figure 1:**
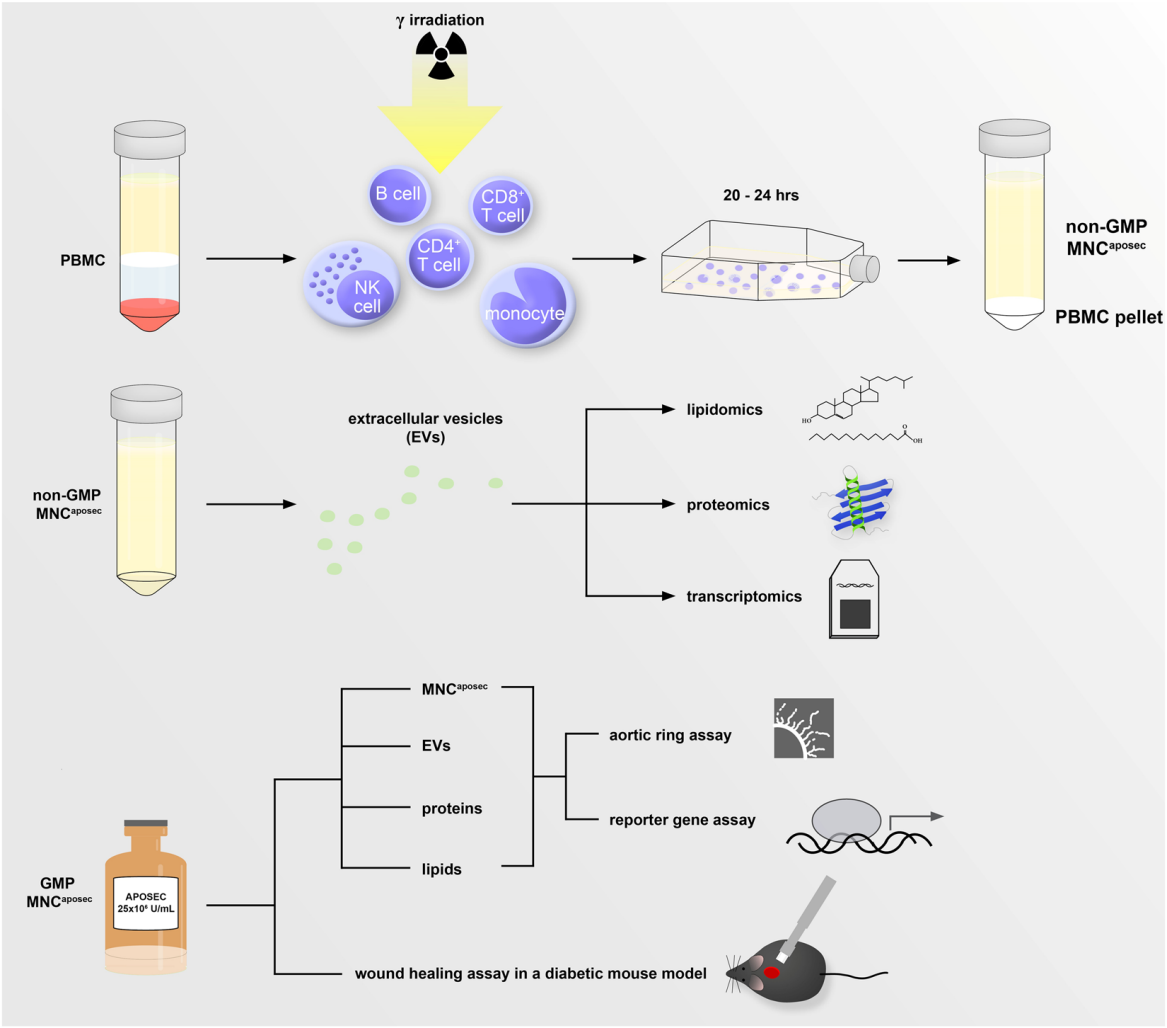
Generation and analysis of MNC^aposec^. (**A**) Isolated PBMCs, consisting primarily of T cells, B cells, NK cells, and monocytes, were isolated by Ficoll density gradient centrifugation. To obtain MNC^aposec^, cells were γ-irradiated with 60 Gy and cultured for 20–24 hours. (**B**) Extracellular vesicles (EVs) were isolated from MNC^aposec^ and analyzed by lipidomics, proteomics, and transcriptomics. (**C**) For functional assessment, MNC^aposec^ produced according to Good Manufacturing Practice (GMP) and purified subfractions (EVs, proteins, lipids) were used for *ex vivo* endothelial sprouting assays and promotor activity assays. In addition, the wound healing properties of MNC^aposec^ were investigated in a diabetic mouse model.

**Figure 2:**
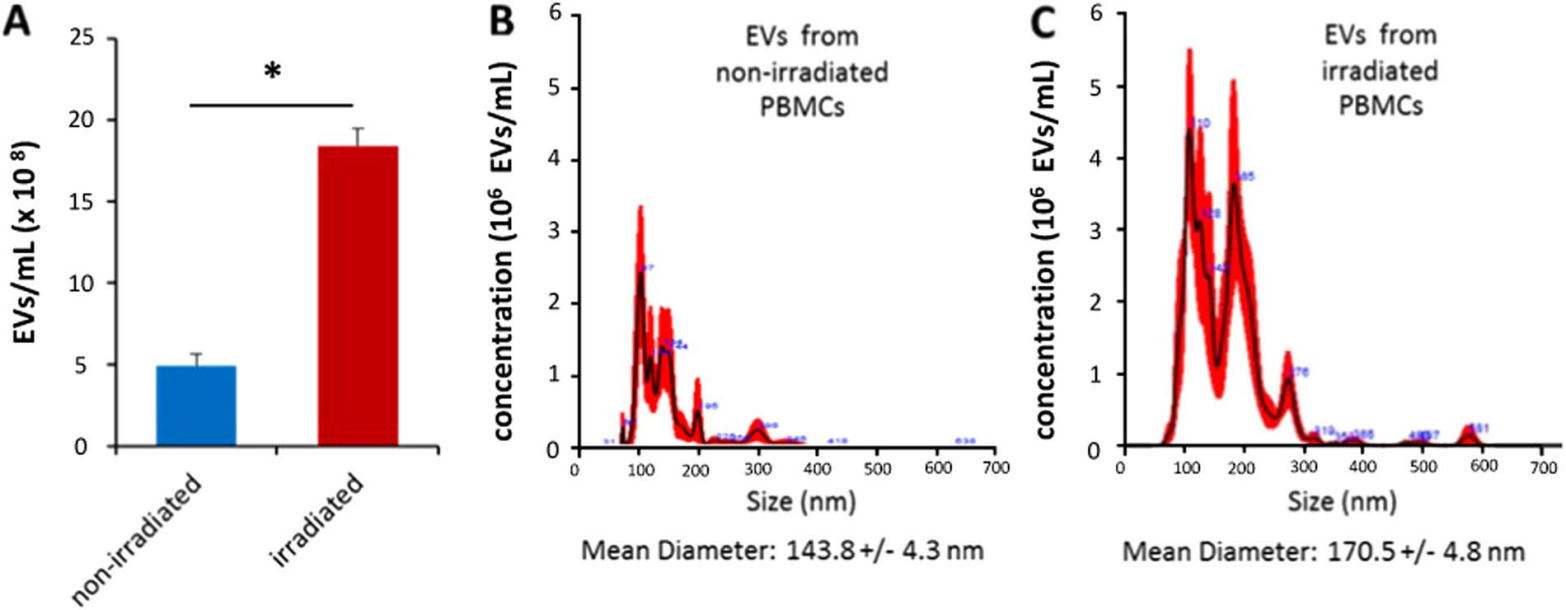
Irradiation changes the number and size of EVs derived from PBMCs. (**A**) Quantification and (**B,C**) size determination of EVs from PBMCs cultured at a concentration of 25 × 10^6^ cells/ml by NanoSight analysis. Irradiation of PBMCs enhanced the release of EVs and generated different vesicle species compared to non-irradiated cells. *p < 0.05.

We previously showed that the secretome of γ-irradiated PBMCs contains EVs, which had wound healing properties in *in vitro* cell culture experiments on epithelial and mesenchymal cells^41^. Therefore, we aimed to investigate in detail the amount, size, and composition of MNC^aposec^-derived EVs. Figure 1 shows the production of the secretome (Fig. 1A), the analytical procedures performed (Fig. 1B), and the functional tests carried out (Fig. 1C). Analysis of the size and number of EVs released from irradiated and non-irradiated EVs by NanoSight technology revealed a 4-fold higher concentration of EVs derived from irradiated PBMCs compared to non-irradiated cells (Fig. 2A). The size of EVs from both irradiated and non-irradiated PBMCs ranged from 80 to 280 nm, but the mean diameter of vesicles derived from irradiated PBMCs was significantly larger than the diameter of vesicles released by non-irradiated PBMCs (170.5 ± 4.8 nm vs. 143.8 ± 4.3 nm; Fig. 2B and C), suggesting that different vesicle species are secreted by cells cultivated under stressed and non-stressed conditions.

### γ-irradiation induces the release of EV-packed proteins involved in intracellular trafficking, immunomodulation, and wound healing

**Figure 3:**
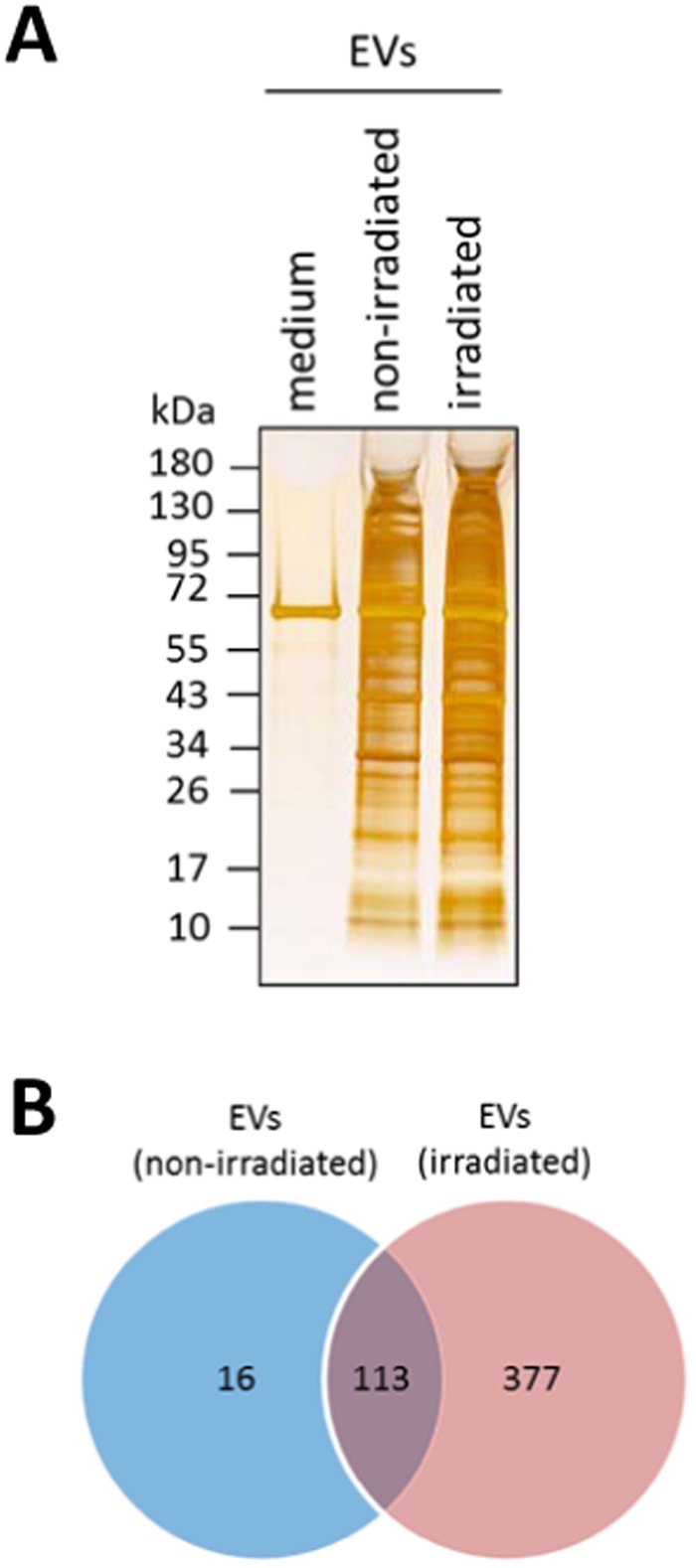
Irradiation changes the protein content of PBMC-derived EVs. (**A**) Representative image of silver staining showed that EVs derived from irradiated and non-irradiated PBMCs comprise different proteins with small to high molecular masses. (**B**) As depicted in the Venn diagram, proteome analysis revealed a higher number of proteins in EVs from irradiated PBMCs than in EVs from non-irradiated PBMCs.

To investigate the protein content of EVs, we cultured irradiated PBMCs from 10 donors in EV-free (pre-centrifuged) medium. After 24 hours, the EVs were harvested, pooled, and the protein content analyzed by SDS-PAGE and HPLC-MS. Silver staining revealed a single band at approximately 67 kDa, corresponding to human serum albumin, in the EV-free medium (Fig. 3A). Uncropped micrographs are available at Supplementary Fig. S1. In contrast, EVs released by irradiated and non-irradiated PBMCs presented numerous proteins of various molecular weights. Because the quantitation of EVs derived from irradiated and non-irradiated cells by silver staining was comparable, we investigated whether they differ qualitatively using a whole proteomics approach. A total of 129 proteins were detected in EVs derived from non-irradiated PBMCs, while 490 secreted proteins were found in EVs after γ-irradiation (Fig. 3B). All proteins identified in the different EVs are listed in Tables S1–S3. Interestingly, almost all proteins released by non-irradiated cells were also present in irradiated PBMCs. Only 16 proteins were exclusively detected in EVs from non-irradiated PBMCs. In contrast, 377 proteins were specifically present in EVs of irradiated PBMCs, suggesting that γ-irradiation significantly changes vesicle formation and/or packaging.

To investigate the possible biological functions of the proteins identified in EVs from irradiated PBMCs, Gene Ontology functional classification was performed. The most significant processes were related to vesicle formation, trafficking, immunological processes, and wound healing (Table 1).

**Table 1:** Gene Ontology functional classification of proteins identified in EVs from irradiated PBMCs.^+^, ^*^ and ^§^ denote biological processes related to vesicle formation and transportation, wound healing, and immunological processes, respectively.

GO Biological Process	*Homo sapiens*	#	Expected	Fold Enrichment	*P*-value
Cell activation*	1029	162	24.30	6.67	1.76E-81
Exocytosis^+^	794	144	18.75	7.68	8.00E-79
Transport^+^	4446	299	105.01	2.85	5.82E-77
Vesicle-mediated transport^+^	1921	203	45.37	4.47	2.08E-76
Cell activation involved in immune responses ^§^	611	128	14.43	8.87	5.29E-76
Leukocyte activation involved in immune response ^§^	607	127	14.34	8.86	2.60E-75
Secretion by cell^+^	997	150	23.55	6.37	2.08E-72
Leukocyte mediated immunity ^§^	745	129	17.60	7.33	7.16E-68
Positive regulation of immune system process ^§^	1073	104	25.34	4.10	2.45E-33
Positive regulation of immune response ^§^	783	79	18.49	4.27	3.45E-26
Endocytosis^+^	660	64	15.59	4.11	1.94E-20
Signal transduction*	5193	217	122.66	1.77	9.14E-20
Blood coagulation*	296	41	6.99	5.86	2.96E-18
Platelet activation*	143	30	3.38	8.88	5.65E-18
Cell communication^+^	5692	224	134.44	1.67	2.76E-17
Wound healing*	471	49	11.12	4.40	6.05E-17
Response to wounding*	562	52	13.27	3.92	5.73E-16
Cell-cell adhesion*	448	45	10.58	4.25	3.92E-15

### EVs from irradiated PBMCs contain a variety of bioactive phospholipids

**Figure 4:**
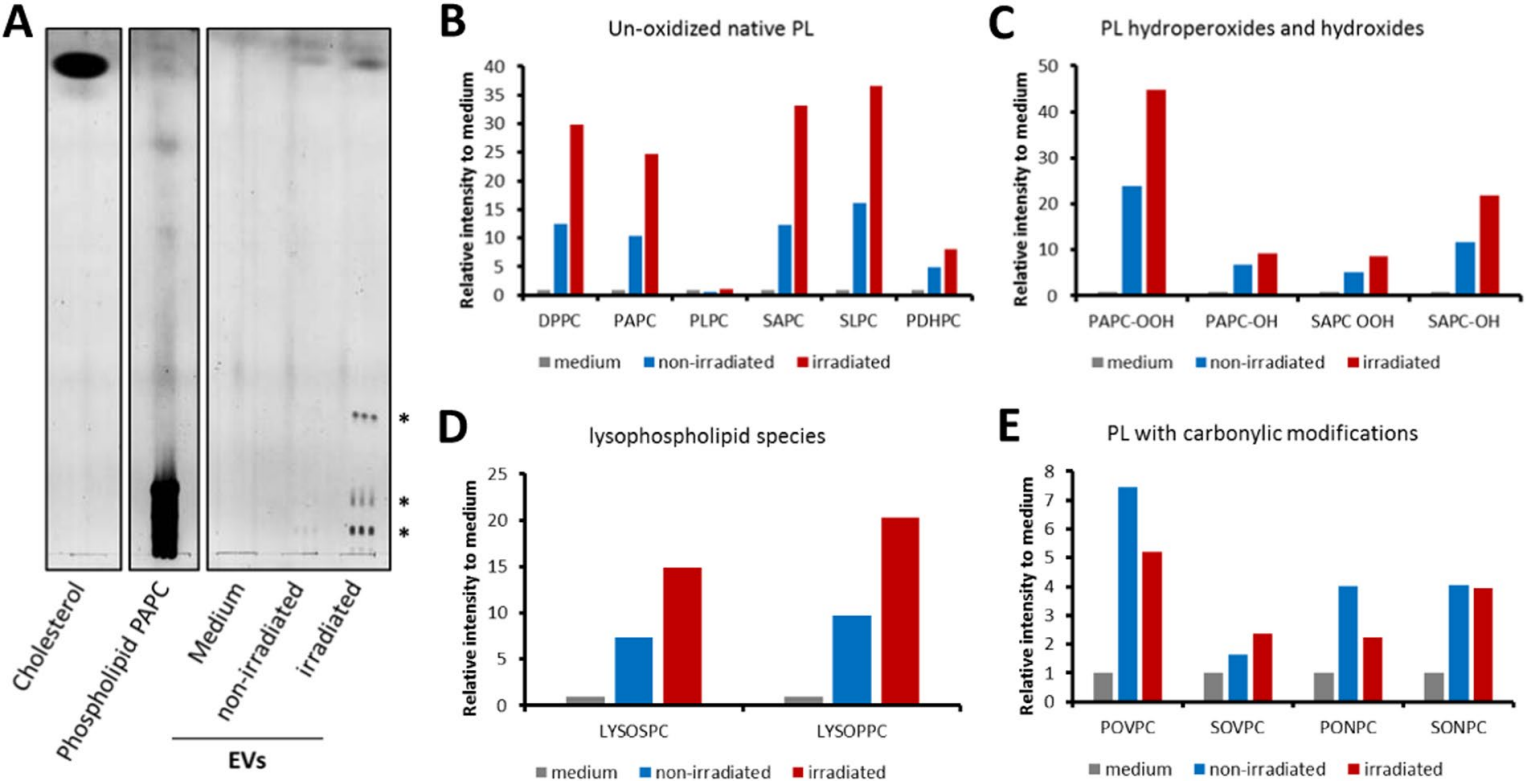
EVs from irradiated PBMCs contain different lipid species. (**A**) Thin layer chromatography (TLC) revealed higher concentrations of polar lipid species co-migrating with phospholipids and cholesterol in EVs from irradiated PBMCs compared to EVs from non-irradiated PBMCs. Standards for cholesterol and partially oxidized 1-palmitoyl-2-arachidonoyl-sn-glycero-3-phosphocholine are shown on the left and were run simultaneously with other samples on the same gel. Asterisks (*) indicate different polar lipid species. (**B-E**) To investigate the phospholipid content of EVs in more detail, LC-MS/MS was performed with pooled PBMCs from 10 donors. EVs from irradiated PBMCs contained un-oxidized phospholipid, oxidized phospholipid (-OOH, -OH), lysophospholipid species, and carbonyl phospholipid species. Irradiation induced higher amounts of different un-oxidized phospholipid, oxidized phospholipid (-OOH, -OH), and lysophospholipid species in EVs.

In order to compare the prevalence of different lipid species in EVs from irradiated and non-irradiated PBMCs, we performed thin layer chromatography (TLC). EVs released by PBMCs predominantly contained cholesterol, followed by phospholipids, according to their co-migration with corresponding standards (Fig. 4A). Full silica gel is available at Supplementary Fig. S2. Irradiation of PBMCs strongly increased EV release and the amount of detectable lipids. Detailed analysis of the phospholipids by liquid chromatography-mass spectrometry (LC-MS/MS) indicated that the EVs contained a variety of native and oxidized bioactive phospholipids (Fig. 4B–E). We observed that the amount of phospholipids was increased in the EV preparation from irradiated PBMCs compared to non-irradiated PBMCs (Fig. 4B–E), corroborating our TLC findings (Fig. 4A). The major phosphocholine species detected in the EV lipid preparations by our screening method^42^ were 1,2-dipalmitoyl-*sn*-glycero-3-phosphocholine (DPPC), 1-palmitoyl-2-arachidonoyl-*sn*-glycero-3-phosphocholine (PAPC), 1-stearoyl-2-arachidonoyl-*sn*-glycero-3-phosphocholine (SAPC), and 1-stearoyl-2-linoleoyl-*sn*-glycero-3-phosphocholine (SLPC) (Fig. 4B and C). The relative amount of 1-palmitoyl-2-linoleoyl-*sn*-glycero-3-phosphocholine (PLPC) in EVs could not be determined because its signal in the growth medium lipid extract control introduced high background.

Next, we analyzed whether the EVs contain oxidation products of the unsaturated phospholipids. Oxidized phospholipids have emerged as versatile signaling compounds^43^ and can be secreted from cells similar to protein signaling compounds in the regulation of inflammation^44^. The EVs contained the hydroperoxides and hydroxides (-OH, -OOH; Fig. 4C) of the major unsaturated phospholipids, but also the carbonyl-containing and potentially reactive phosphocholine species (POVPC, PONPC; Fig. 4D), which can be taken up by cells, resulting in cell signaling and protein modification^45^.

### γ-irradiated PBMCs exhibit a distinct miRNA secretion signature in EVs

**Figure 5:**
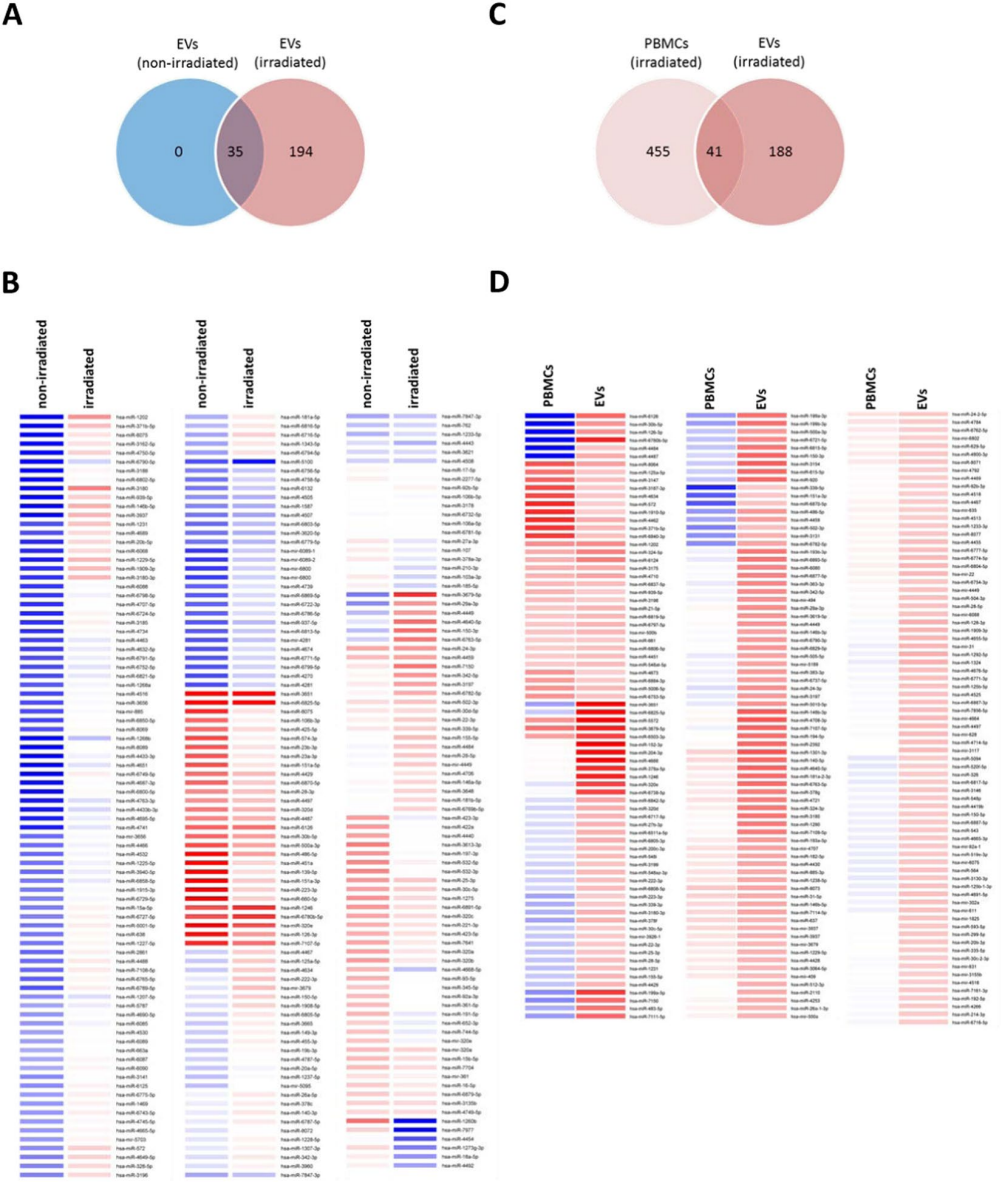
Irradiation changes the miRNA content in EVs. To examine the miRNA content of EVs and PBMCs, PBMCs from 10 donors were cultivated in EV-free (pre-centrifuged) medium. (**A**) Venn diagram showing the number of identified miRNAs. (**B**) Heat map analysis depicted the up-regulation (red) and down-regulation (blue) of miRNAs in EVs derived from irradiated vs. non-irradiated PBMCs. (**D**) As in (**C**) but PBMCs vs. EVs after irradiation.

EVs reportedly function as cargo for miRNA from donor cells to recipient cells, mediating communication between different cell types^46,47^. Therefore, we investigated the miRNA composition of EVs and whether γ-irradiation affected the miRNA content of EVs. RNA length profile analysis revealed that EVs carried short RNA fragments up to 80 nucleotides (Fig. S3), mainly representing miRNAs (20–25 nucleotides), whereas PBMCs had RNAs from the entire spectrum of length. In order to investigate the miRNA expression profile of EVs and PBMCs, RNA was isolated and analyzed using an Affymetrix miRNA 4.0 Array. γ-Irradiation significantly changed the miRNA composition in EVs (Fig. 5A and B). Of the 229 miRNAs detected in EVs from γ-irradiated PBMCs, only 35 (Tables S4 and S5) were also present in EVs derived from non-irradiated PBMCs, suggesting a strong influence of γ-irradiation on miRNA packaging into EVs. When we compared the miRNA content of γ-irradiated PBMCs to the content of EVs derived from the same cells, we found that a high number of all detected EV-derived miRNAs (188 out of 229; 82%) (Table S6) were exclusively present in EVs, suggesting enrichment of certain miRNAs in EVs. In contrast, a rather small number of detectable miRNAs in PBMCs (41 out of 455; 9%) (Table S7) were transferred to EVs (Fig. 5C, Table S8). The heat map in Fig. 5D shows a quantitative comparison of the 229 miRNA detected in EVs from γ-irradiated PBMCs and the donor cells. These data indicate that γ-irradiation strongly affects the miRNA composition of EVs, which could significantly contribute to its regenerative activity.

### MNC^aposec^ exhibits angiogenesis-promoting features

**Figure 6:**
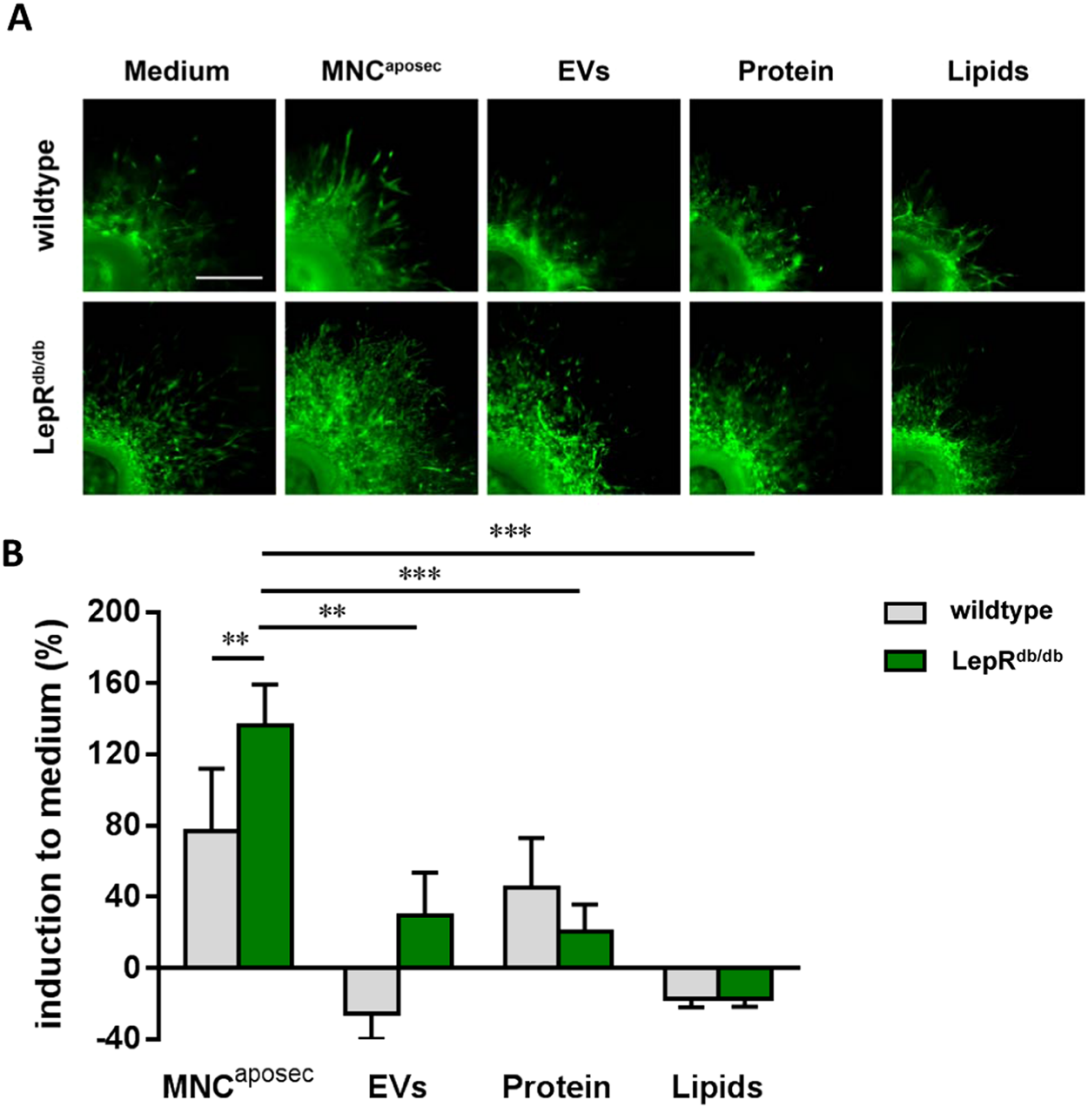
MNC^aposec^ enhances vessel sprouting in *ex vivo* aortic rings obtained from wildtype and diabetic mice. (**A**) Representative immunofluorescent micrographs of aortic rings from wildtype and diabetic (LepR^db/db^) mice (n = 5 per group) after 5 days culture under the indicated conditions (Medium, MNC^aposec^, EVs, proteins, and lipids). The fractions were purified from MNC^aposec^ produced under GMP conditions. Aortic rings were treated with MNC^aposec^ and the purified fractions corresponded to 4 × 10^6^ PBMCs. Scale bar = 500 µm. (**B**) Area of vessel sprouting under the different conditions and settings relative to the medium control group. MNC^aposec^ -induced sprouting of endothelial cells was significantly stronger in aortic rings from LepR^db/db^ mice than those from wildtype mice. Endothelial cell sprouting was significantly stronger when cultured with the whole secretome (MNC^aposec^) compared to the purified fractions. Data are means +/− SD. Mean represents statistical analysis of three donors; **p < 0.01; ***p < 0.001.

We previously demonstrated that MNC^aposec^ exhibits strong angiogenic potential *in vitro*^24^ and in mouse and porcine wound models *in vivo*, and was associated with accelerated wound healing^26,27^. In addition, we demonstrated that purified small EVs were more potent in inducing keratinocyte and fibroblast migration in *in vitro* scratch assays^41^, and recent publications have suggested that small EVs are key players in neo-angiogenic processes^35–39^. Therefore, we investigated whether purified EVs derived from irradiated PBMCs also demonstrate angiogenic potential in an *ex vivo* aortic ring assay and compared them to the protein and lipid fraction, as well as the whole secretome. To address the question of whether the pro-angiogenic effects can also be achieved in a diabetic setting, we additionally analyzed and compared vessel sprouting by aortas obtained from wildtype and diabetic (LepR^db/db^) mice. The effective dose of MNC^aposec^ was titrated to a concentration of 4 × 10^6^ PBMCs/ml (Fig. S4). Though MNC^aposec^ strongly induced sprouting in all of the aortic rings tested, the pro-angiogenic effect was even more pronounced in aortas obtained from diabetic mice (Fig. 6).

**Figure 7:**
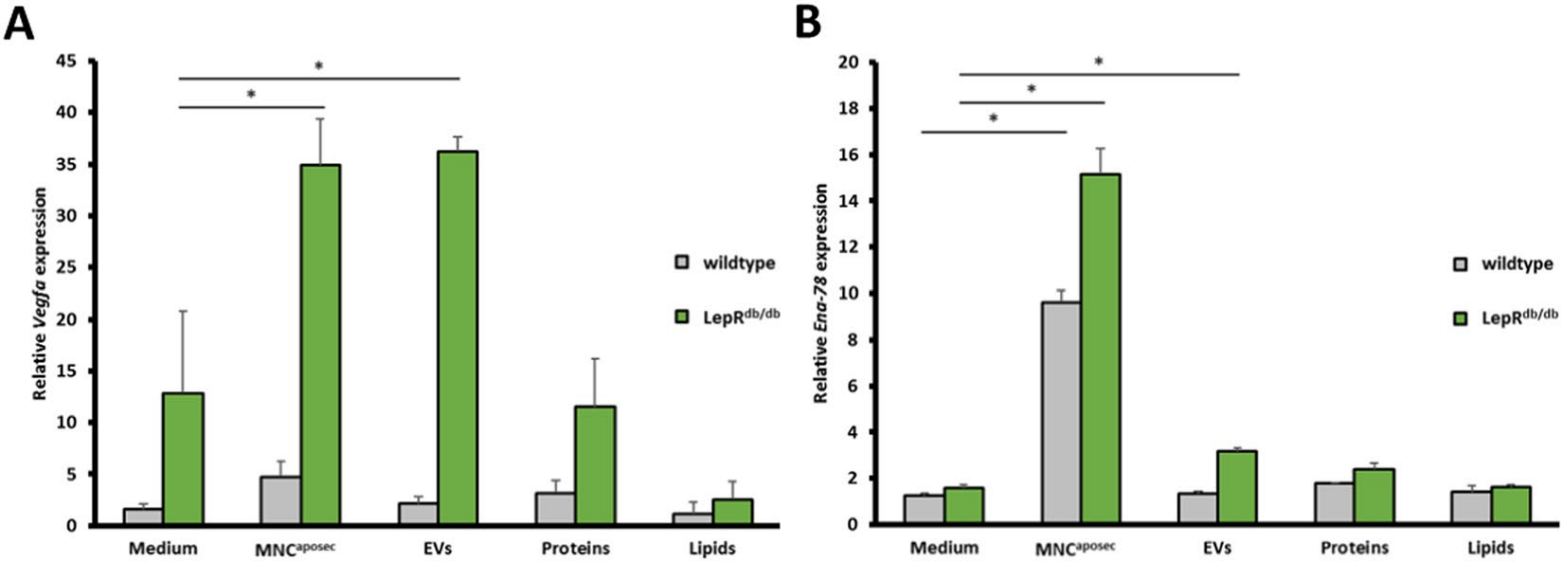
MNC^aposec^ and EVs induce *Vegfa* and *Ena-78* mRNA expression in aortas from diabetic mice. Aortic rings were stimulated with medium, MNC^aposec^, EVs, proteins, and lipids and (**A**) *Vegfa* and (**B**) *Ena-78* expression normalized to the housekeeping gene *Gapdh*. Data are means +/− SD. Mean represents statistical analysis of three replicates; *p < 0.05.

Next, we investigated which component(s) of the secretome (EVs, proteins, lipids) conferred angiogenesis-promoting properties. The whole secretome had the highest efficiency in inducing vessel sprouting when compared to subfractions (Fig. 6). Though proteins increased sprouting by 45% and 20% in aortic rings from wildtype and diabetic mice, respectively, an induction of vessel sprouting by EVs was only observed in diabetic mice (30% increased sprouting compared to medium alone). Lipids failed to induce vessel sprouting in any setting tested. These data indicate that the full angiogenic potential of MNC^aposec^ is only achieved by applying the whole secretome. In line with the pro-angiogenic effect of MNC^aposec^ and the EV fraction in the aortic ring assay, *Vegfa* and *Ena-78* mRNA expression was induced in aortas from diabetic animals, suggesting a distinct pro-angiogenic response in a hyperglycemic system (Fig. 7)

**Figure 8:**
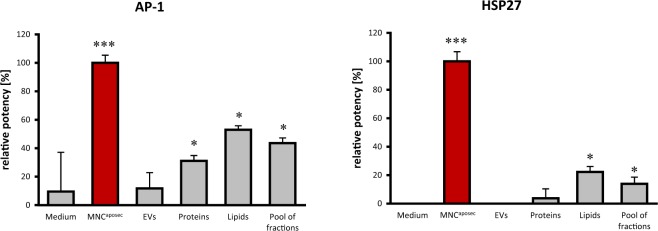
Subfractions of MNC^aposec^ fail to exert synergistic effects on AP-1 promotor activity and phosphorylation of HSP27. To investigate the effect of MNC^aposec^ and the purified fractions on signaling cascades involved in angiogenesis and cell survival, we performed reporter gene assays of (**A**) AP-1 and (**B**) Hsp27. MNC^aposec^ exhibited the strongest induction of AP-1 promotor activity and phosphorylation of HSP-27. Lipids and proteins were able to activate AP-1, but only the lipid fraction induced phosphorylation of HSP27. Furthermore, no additive effect of pooled EVs, proteins, and lipids was detected in any assay performed. Data are means +/− SD. Mean represents statistical analysis of three replicates; *p < 0.05, ***p < 0.001.

We also investigated whether gene activation is involved in the pro-angiogenic activities of MNC^aposec^ and the purified fractions. As the involvement of activator protein 1 (AP-1) in angiogenic processes is well documented^48^, we chose an AP-1 reporter gene assay to address this question. As expected, we observed a strong induction of AP-1 promotor activity after the addition of MNC^aposec^ (Fig. 8A). The protein and lipid fraction significantly induced the AP-1 promoter by 35% and 55%, respectively. Interestingly, EVs had no effect on the activity of the AP-1 promotor, suggesting that they act most likely via repression of gene expression by miRNAs. As the mitogen-activated protein (MAP) kinase pathways involving heat shock protein 27 (HSP27) have also been shown to be crucial for wound healing^49,50^, we established an HSP27 potency assay and investigated the effects of EVs, proteins, and lipids on this wound healing-associated signaling pathway. Corroborating the findings of the AP-1 promotor assay, HSP27 phosphorylation was strongly induced by the whole secretome. The lipid fraction exhibited weak activity (~20%) compared to MNC^aposec^, and EVs and proteins exhibited little to no effect (Fig. 8B). Pooling subfractions to generate MNCaposec failed to display an additive effect of EVs, proteins, and lipids, suggesting the presence of further bioactive molecules secreted by γ-irradiated PBMC.

### MNC^aposec^ accelerates wound healing in LepR^db/db^ mice

As all subfractions of MNC^aposec^ demonstrated only weak activities in our *ex vivo* and *in vitro* potency assays, we evaluated the effects of MNC^aposec^ on diabetic wound healing. We excised full-thickness skin wounds on the mid-dorsum of LepR^db/db^ mice, followed by topical administration of MNC^aposec^ or drug vehicle. Compared to control mice, wound closure was accelerated in MNC^aposec^-treated LepR^db/db^ mice, as indicated by smaller wound circumferences on days 11, 14, 18, and 25 post-wounding (Fig. S5A,B). On day 25 post-wounding, mice were sacrificed and wound morphology was investigated. H&E staining revealed a significantly smaller wound area in MNC^aposec^-treated mice compared to drug vehicle-treated control group (Fig. S5C), indicating that MNC^aposec^ ameliorates diabetic wound healing. In contrast to our observations in other wound models (*i.e*., full-thickness skin wounds in wildtype mice and burn wounds in pigs^26,27^), where blood vessel formation was increased after MNC^aposec^ treatment, CD31 staining of blood vessels remained unaffected by MNC^aposec^ treatment in the present study (Fig. S5D,E).

## Discussion

For a long time, cell-based approaches have been considered the promising therapeutic future for promoting tissue regeneration of almost every damaged organ^51,52^. Although this idea was supported by a large number of *in vitro* cell culture and *in vivo* animal experiments, there was considerable disappointment when clinical trials in humans revealed only minor effects or failed^7^. Gnecchi *et al*. were the first group to report that concentrated paracrine factors derived from mesenchymal stem cells are responsible for the tissue regenerative effect in an experimental model of acute myocardial infarction^10^. Since then, numerous studies have further corroborated this finding, partly clarified the biology of paracrine mechanisms and identified the putative underlying mechanisms of secretome-based tissue regeneration^40,53,54^. We reported for the first time that regenerative secretomes consist primarily of proteins, lipids, and EVs, and much effort has been made to identify the factor(s) or subfraction(s) responsible for *in vitro* activities^41^. In particular, the contribution of small EVs (containing exosomes) to regenerative processes, including wound healing, has been investigated extensively^35–39^. In line with these investigations, we recently demonstrated in *in vitro* scratch wound assays of human keratinocyte and fibroblast cultures that MNC^aposec^-derived EVs accelerated wound healing processes^41^.

Using a multi-omics approach, we analyzed the constituents of PBMC-derived EVs and their altered composition after γ-irradiation. Recent studies have reported that certain stimuli affect vesicle formation processes, increasing the release of EVs^55^. In line with these data, we demonstrated that γ-irradiation augments the release of EVs from PBMCs. Moreover, we provide evidence that PBMCs dynamically fine-tune lipid, protein, and miRNA species packaged and released in EVs in response to radiation. As EVs shuttle biomolecules from one cell to the other, γ-irradiation is able to influence functional properties of non-irradiated recipient cells. Our multi-omics analyses showed that the protein, lipid, and miRNA composition of secreted EVs shifts towards a regenerative phenotype upon irradiation. Interestingly, comparative proteome analysis of EVs derived from irradiated and non-irradiated cells revealed 377 proteins that were exclusively found in EVs from irradiated PBMCs compared to non-irradiated counterparts. *E.g*. galectin-1^56^, serpin B9^56^, lysozym C^57^, HLA class I antigen, leukocyte elastase inhibitor^58^, leukotriene A-4 hydrolase^59^, proteins reportedly involved in immunological processes, were secreted in EVs by irradiated PBMCs. We furthermore detected proteins predominantly associated with wound healing, such as calreticulin^60^, fibrinogen α/β/γ chains^32^, platelet factor 4^61^, and platelet glycoproteins^62^. We therefore hypothesize that the tissue regenerative effect attributed to irradiated PBMC-derived EVs is, at least in part, due to selected proteins packed and secreted in EVs. Delineating their specific functions merits future investigations.

The miRNA composition of secreted vesicular EVs changed remarkably after γ-irradiation. Intriguingly, we found that a large number of miRNAs were exclusively present in EVs derived from irradiated PBMCs. The majority of detected miRNAs have not yet been well characterized, making data interpretation delicate. Nevertheless, our results suggest that at least some of these miRNAs are associated with regenerative processes; thus, further studies are needed to clarify their contribution to wound healing. For example, we detected several miRNAs reportedly associated with major wound healing events. One of the best characterized miRNAs present in EVs from γ-irradiated PBMCs was miRNA-21, which has been shown to be upregulated in the skin after injury and to contribute to wound healing by enhancing keratinocyte, fibroblast, and endothelial cell migration, wound contraction, collagen deposition, and angiogenesis^35,63^. In addition, we detected miRNA-126^64–66^, miRNA-30^67^, miRNA-31^68,69^, and miRNA-335, all involved in angiogenic and skin wound healing events, even in a diabetic setting^70^, suggesting that γ-irradiation promotes the release of vesicular miRNAs with tissue regenerative capacity. Intriguingly, purified EVs, containing miRNAs, showed no or only minor effects on vessel sprouting in wildtype- and diabetic mice- derived aortic rings, respectively, suggesting that the main regenerative function of EVs is not dedicated to the induction of endothelial cell proliferation. This discrepancy could be explained by the fact that the majority of studies investigating pro-angiogenic potential of miRNA apply high doses of purified miRNAs without EV packaging.

As bioactive oxidized phospholipids play a crucial role in two critical phases of wound healing, the sensing of cell damage patterns^71^ by the innate immune system, and the resolution phase of inflammation^72^, we analyzed the phospholipid content of EVs released by non-irradiated and γ-irradiated PBMCs. Compared to EVs from non-irradiated PBMCs, EVs from γ-irradiated PBMCs contained high amounts of bioactive phospholipids. The altered lipid composition in EVs after irradiation was comparable to our previous study on soluble lipids in MNC^aposec^^41^. PAPC-OOH was enriched in EVs from irradiated PBMCs and has recently been implicated in dendritic cell maturation and differentiation^73^, suggesting that oxidized lipids play a role in shaping the immune response rather than inducing angiogenesis or proliferation and the migration of skin cells as shown previously^41^. Furthermore, lysophosphatidylcholines, a phospholipid species highly abundant in EVs derived from stressed PBMCs, are important precursors of lysophosphatidic acid, a major factor in tissue remodeling and immune regulation during wound healing^74^. Notably, the wound fluid from diabetic rat wounds differs in lysophospholipid composition from controls^75^, suggesting a potential application of PBMC secretome. Finally, POVPC were detected in the EVs from PBMCs after γ-irradiation. This carbonyl-containing, fragmented, oxidized lipid can induce inflammasome activation^76^, which has been shown to accelerate the healing of skin wounds^77^. Tentatively, our results indicate that irradiated PBMCs contribute to wound healing, at least partially, by releasing distinct bioactive phospholipid species in EVs.

In a previous study, we found that the EV fraction exerted proliferative and stimulatory effects *in vitro*^41^, and a growing number of studies have focused on EV-based therapies in wound healing. Therefore, we addressed whether MNC^aposec^-derived EVs may solely be responsible for skin wound regeneration. In wound healing settings, numerous studies have attributed pro-angiogenic capacities to stem cell-derived EVs, and various underlying mechanisms have been identified thus far (30–34). In contrast to the existing literature on MSC-derived EVs, we observed diminished sprout-inducing activity in *ex vivo* aortic rings and reduced activation of AP-1 and HSP27 with the EV-enriched fraction compared to MNC^aposec^. These data suggest that EVs released by stem cells, PBMCs, or other cells have distinct properties with regard to angiogenesis. Pro-angiogenic activity, activation of the transcription factor AP-1, and HSP27 phosphorylation were remarkably compromised in all subfractions tested. Though secretome-derived proteins exhibited a moderate pro-angiogenic effect, the lipid subfraction failed to induce endothelial cell sprouting in aortic rings obtained from wildtype and diabetic mice. However, the pro-angiogenic effect of EVs derived from MNC^aposec^ was only observed in the diabetic setting. The pro-angiogenic factor VEGFA and wound healing-promoting factor Ena-78 were strongly induced by these EVs in diabetic aortas, but not in wildtype aortas. These findings suggest that different mechanisms account for the pro-angiogenic activity in the two settings. More sophisticated experiments are needed to fully elucidate the signaling pathways involved in diabetic and physiological wound healing events.

In addition, the combination of these three subfractions did not lead to a synergistic effect on AP-1 promoter activity and phosphorylation of HSP27, suggesting that other pro-angiogenic components are present in MNC^aposec^. Currently we are investigating the contribution of free nucleic acids, present in the secretome (data not shown), to the pro-angiogenic effect of MNC^aposec^. Together, our data suggest that secretome subfractions concertedly confer tissue-repairing characteristics. Based on the data presented here, we recommend the use of complete secretomes rather than apportioned constituents for clinical wound management. However, since we only investigated neo-angiogenesis in the present study, we cannot rule out that other important regenerative properties of secretome-promoted wound healing are attributed solely to EVs. Further *in vivo* wound healing studies with secretome subfractions have to be conducted to gain additional functional insights into their mechanism and contribution to tissue regeneration.

Having demonstrated that the whole cell secretome is necessary for full regenerative capacity, we applied MNC^aposec^ to full-thickness skin wounds in a hyperglycemic mouse model (LepR^db/db^) to investigate whether MNC^aposec^ represents a promising therapeutic option for non-healing diabetic foot ulcers. In accordance with previous observations from wildtype mouse models^26^ and a porcine burn model^27^, MNC^aposec^ also accelerated wound closure in LepR^db/db^ mice. In contrast to these models^26,27^, where pro-angiogenic effects were observed during the early phase of wound healing, we detected no difference in blood vessel formation in MNC^aposec^-treated LepR^db/db^ compared to wildtype 25 days after wounding. Assessment of angiogenesis-related events at earlier time points has to be conducted to fully elucidate the pro-angiogenic potential of MNC^aposec^ in diabetic mice.

Although we could demonstrate less angiogenic potential of single purified subfractions of cellular secretomes compared to the whole secretome, our study has some limitations. Though we have shown that the use of human secretomes in rodents is harmless and efficient in several previous studies^28^, potential xenogenic influences cannot be completely ruled out in our diabetic mouse model. Further experiments with allogenic MNC^aposec^ will deepen our understanding of the regenerative action of MNC^aposec^ and its subfractions in diabetic wound healing. For consistency with the medicinal product used in clinical studies in humans, we pooled secretomes and subfractions from 10 donors. We were therefore not able to determine possible donor-to-donor variations in the present study.

In conclusion, our study provides encouraging data that have paved the way for a clinical trial testing the efficacy of allogenic MNC^aposec^ on non-healing diabetic foot ulcers. As MNC^aposec^ has already passed phase I safety trials, patient recruitment for phase II is already in progress.

## Materials and Methods

### Ethics statements

Human leukocyte concentrates were obtained from healthy volunteers as a byproduct of the thrombocyte donation procedure. This study was approved by the local ethics committee at the Medical University of Vienna (vote: EK No: 1326/2013). All donors provided informed written consent and demographic data of individual donors are listed in Table S9. Animal experiments were approved by the Animal Protocol Review Board of the City Government of Vienna, Austria (vote: 190097/2015/9) and were performed in accordance with the Austrian guidelines for the use and care of laboratory animals. All methods were performed in accordance with relevant guidelines and applicable regulations.

### Generation of the secretome of human PBMCs

PBMCs were isolated as described previously^27,41^. After isolation, cells were resuspended in serum-free CellGenix culture medium (Freiburg, Germany) at a density of 25 × 10^6^ cells/ml and γ-irradiated with 60 Gy. After 20 hours of incubation, cell suspensions were centrifuged to eliminate cells and cellular debris and passed through a 0.2-µm pore filter (Whatman, GE Healthcare, Little Chalfont, UK). In anticipation of potential clinical use, the secretome of irradiated PBMCs was also produced according to GMP guidelines as described previously^24^. To reduce high donor-to-donor variability, pools of 10 donors were used (Table S10). Concentrations of interleukin 8 (Il-8) and transforming growth factor beta 1 (TGF-β1) were assessed in MNC^aposec^ of individual donors and pools of MNC^aposec^ and revealed smaller variability between pooled samples compared to individual samples (Table S11). To reduce donor-to-donor variability, pooled samples were used for experiments.

### Isolation of MNC^aposec^ components

For functional *in vitro* assays, different components of the MNC^aposec^ were isolated (Fig. S6). Lyophilized GMP MNC^aposec^ was reconstituted in 0.9% NaCl to obtain the initial concentration of 25 × 10^6^ cells/ml. Proteins were precipitated by the addition of polyethylene glycol 4000 (30% w/v; Fluka BioChemika, Sigma-Aldrich) on ice for 15 minutes and centrifuged at 20,000 *g* for 15 minutes at 4 °C. Total lipids were extracted using the chloroform/methanol-assisted Folch method as described previously^78^. EVs were isolated as described below. All fractions were reconstituted in serum-free CellGenix culture medium, corresponding to a concentration of 25 × 10^6^ cells/ml, and stored at −80 °C until further use. To ensure comparability, proteins and lipids were used in the same concentrations as present in MNC^aposec^.

### Isolation of EVs and NanoSight analysis

EVs were isolated by ultracentrifugation of the secretome of irradiated and non-irradiated human PBMCs. To obtain EVs, secretomes were centrifuged at 20,000 *g* for 20 minutes and at 110,000 *g* for 120 minutes at 4 °C in an SW 41 swinging bucket rotor (Beckman Coulter). For lipid, protein, and RNA analysis, irradiated and non-irradiated PBMCs were cultured in pre-centrifuged (220,000 *g* for 24 hours at 4 °C) serum-free CellGenix to deplete EVs from the culture medium. The absolute number and size of EVs were assessed using a NanoSight, NS500 instrument (Malvern Instruments, Malvern, UK). The nanoparticles were visualized by laser light scattering and the motion of each nanoparticle tracked from frame to frame by NTA3.1 software. Recorded videos were analyzed by the NTA3.1 software. For comparability, isolated EVs were employed in the same concentrations as EVs present in MNC^aposec^.

### RNA isolation and transcriptome analysis

EVs and the corresponding cultured PBMCs were obtained from 10 different donors, pooled, and lysed by peqGOLD TriFast reagent (VWR peqlab, International GmbH, Darmstadt, Germany). Total RNA was extracted following the manufacturer’s instructions. RNA cleanup was performed using the RNeasy MinElute Cleanup Kit (Qiagen, Hilden, Germany). RNA quality was determined on an Agilent 2100 Bioanalyzer (Agilent, Böblingen, Germany) and expression profiling performed with an Affymetrix miRNA 4.0 Array (Affymetrix part of Thermo Fisher Scientific, Inc.). Affymetrix GeneChip analysis was carried out at the Genomics Core Facility at the Medical University of Vienna. Data were analyzed using GeneSpring Version 15.0 software (Agilent, Santa Clara, California).

### Proteome analysis

EVs released by irradiated and non-irradiated PBMCs were lysed with Laemmli sample buffer (BioRad Laboratories, Hercules, California, USA) containing 0.1 M DL-dithiothreitol (Sigma-Aldrich). After sonication, equal amounts of lysates were separated by SDS-PAGE and silver stained as described elsewhere^2^. For proteomics analysis, total exosomal RNA and Protein Isolation Kit (Invitrogen, Thermo Fisher Scientific, Inc., Waltham, Massachusetts, USA) was used to isolate proteins from EVs from irradiated and non-irradiated PBMCs (pool of 3 donors). Proteome analysis was performed at the Proteomics Core Facility at the Medical University of Vienna. Briefly, mass spectrometric detection and MS/MS analysis was performed using the Q-Exactive Orbitrap BioPharma (ThermoFisher, Bremen, Germany). Peptides were introduced into the nano electrospray source and the ionization was performed using a steel needle with 20 µm inner diameter and 10 µm tip. Needle voltage was set to 2 kV in positive mode and the top 10 ions were selected for MS/MS analysis. Resolution was set to 70.000 for full MS scans, ions with single charge were excluded from MS/MS analysis and fragmented ions were excluded for 60 seconds from further fragmentation. Generated raw files were converted into mgf by applying MS Convert (http://proteowizard.sourceforge.net/tools.shtml). Database search (SwissProt_human, version from November 2017)) was performed by submitting the mgf files to MASCOT v. 2.6.0 (Matrix Science, London, UK) through ProteinScape (Bruker, Bremen, Germany) using following parameters: Taxonomy: Homo sapiens; Modifications: carbamidomethyl on C as fixed, deamidation on N and Q, carboxymethylation on M and phosphorylation S and T as variable; Peptide tolerance was set to 20 ppm and the MS/MS tolerance to 0.05 Da; Trypsin was selected as the enzyme used; False discovery rate (FDR) was set to 1% and the decoy database search was used for estimating the FDR; The Mascot search results were further refined by applying an additional data analysis step using Scaffold v4.8.4 (www.proteomesoftware.com).

#### Functional analysis of EV-derived proteins

The WEB-based Gene Stet Analysis Toolkit (WebGestalt) database was used to functionally analyze the 490 proteins found in EVs released by irradiated PBMCs. WebGestalt enables the detection of enrichment of gene ontology (GO) terms in a set of proteins of interest. The whole human genome was used as a reference set for enrichment analysis and the Benjamini-Hochberg method for multiple testing was applied with a significance level of p ≤ 0.05 and FDR < 5%. Biological processes were manually assigned to clusters associated to vesicle formation and transportation, wound healing, and immunological processes, respectively.

### Thin layer chromatography

Total lipids were extracted from EVs from irradiated and non-irradiated PBMCs (pool of 5 donors) cultured in pre-centrifuged serum-free CellGenix medium. Purified EVs were resuspended in 150 µl deionized water and 1350 µl chloroform/methanol (2:1 v/v) supplemented with 0.01% butylated hydroxytoluene (BHT) and aqueous formic acid (1/4 the volume of chloroform/methanol). After centrifugation, the lower organic phase was collected and organic solvents evaporated under an argon stream to avoid oxidation. TLC was performed as described previously^79^.

### Liquid chromatography–mass spectrometry

For phospholipid analysis by mass spectrometry, lipids were isolated from irradiated and non-irradiated PBMCs (pool of 10 donors) as described previously^42^. LC-MS/MS analysis of oxidized and non-oxidized phospholipids was performed at the FTC-Forensisch-Toxikologisches Labor BetriebsgmbH (Vienna) as described previously^42^. High technical reproducibility was verified^42^. Sample peak areas were normalized to the peak areas of the spiked DNPC (internal standard).

### Animal model

C57BL/KsJm/Leptdb (db/db) mice were obtained from Charles River. Twelve-week-old symptomatic diabetic db/db mice were used for the experiments. To determine blood glucose levels, blood was drawn from the tail vein and analyzed using an Akku-Chek Go glucose meter (Roche Diagnostics, Germany). All animals exhibited severely increased blood glucose levels ≥ 300 mg/dl. For skin wounding, the animals’ dorsal hair was shaved and completely removed using a depilatory cream. The skin was cleaned with alcohol and a 1.3 cm full-thickness skin wound excised under aseptic conditions on the mid-dorsum. A total volume of 100 μl of hydrogel matrix containing the therapeutic agent (MNC^aposec^; concentration of 25 × 10^6^ PBMCs/ml) or control drug vehicle (Aqua ad injectabilia, Braun, Melsungen, Germany) was topically administered and the wound covered with Tegaderm™ foil (3M, Maplewood, MN, US). MNC^aposec^ and control were blended with NUGEL (Chemomedica, Austria). Animals received adequate pain management of 0.05 mg/kg buprenorphine (Buprenorphin, Richter Pharma, Austria) immediately and 12 hours after surgery, as well as 0.15 mg/kg Meloxicam (Boehringer, Austria) daily for the first 4 days. Twelve mice were assigned to each group.

### Wound analysis

Three-dimensional wound measurements were performed on days 0, 3, 7, 11, 14, 18, and 25 using a stereoscopic camera (LifeViz micro, Quantificare, France). Stereoscopic wound parameters were evaluated using DermaPix Pro 2.28.5. All parameters were expressed as a percentage of the initial wound parameter after surgery.

### Immunohistochemistry

For immunohistological analysis, tissue samples obtained from diabetic mouse wounds were fixed, paraffin embedded, and cut into 5-μm-thick sections. CD31 staining (1:100, M-20, sc-1506-G, Santa Cruz Biotechnology, Santa Cruz, CA, US) was performed on a Lab Vision Autostainer 360 (Thermo Scientific, Waltham, MA, US). The staining was visualized using Vector NovaRed Peroxidase (VectorLabs, Burlingame, CA, USA). Counterstaining was performed with hematoxylin (Carl Roth GmbH, Karlsruhe, Germany). Images were acquired by an Olympus BX61VS scanning microscope (Tokyo, Japan) and analyzed using the cellSens Imaging Software (Olympus, Tokyo, Japan). Hematoxylin and eosin staining was performed according to a standard protocol.

### Aortic ring assay

Aortic ring assay was performed as described previously^1^ with minor modifications. Murine aortas were excised, cut into rings of approximately 1 mm length and sandwiched between two layers of 2 mg/ml human fibrinogen (EMD Millipore, Darmstadt, Germany), 43.3 µg/ml aprotinin (Sigma-Aldrich), and 0.6 U/ml thrombin (Sigma-Aldrich). After polymerization, the fibrin-aorta-fibrin gel was equilibrated with M199 medium (Gibco by Life Technologies) supplemented with 4 mM L-glutamine (Gibco by Life Technologies), antibiotic solution (100 U penicillin, 100 µg/ml streptomycin; Gibco by Life Technologies), 250 ng/ml amphotericin B (Fisher Bioreagents, Fisher Scientific), and 10% fetal calf serum (PAA Laboratories, Pasching, Austria) for 45 minutes. The medium was then changed to complete M199 medium supplemented with MNC^aposec^ corresponding to 1 × 10^6^ PBMCs, 2 × 10^6^ PBMCs, 4 × 10^6^ PBMCs, and 8 × 10^6^ PBMCs and EVs, proteins, or lipids added at a concentration corresponding to 4 × 10^6^ PBMCs. Aortic rings were incubated for up to 5 days and medium was changed every other day. On day 5, aortas were incubated with calcein (Thermo Fisher Scientific) for 1 hour to label viable sprouting endothelial cells. The outgrowth was assessed using an Olympus IX83 scanning microscope (Tokyo, Japan) with cellSens Imaging Software (Olympus, Tokyo, Japan). The outgrowth area was quantified using ImageJ software.

### Reporter gene assays and potency assays

Reporter gene assays for AP-1 were established at Synlab Pharma Institute AG (Switzerland). Human neuroblastoma SH-SY5Y cells containing an integrated expression cassette that encodes firefly luciferase under the transcriptional control of AP-1 were cultured in Ham’s F12/MEM (50:50; Sigma-Aldrich) supplemented with 2 mM L-glutamine (Sigma-Aldrich), 1x MEM Non-essential Amino Acid Solution (Sigma-Aldrich), 15% fetal bovine serum (Gibco), and 1 µg/ml puromycin (Sigma-Aldrich) or DMEM/F12 + Glutamax (Gibco) supplemented with 10% fetal bovine serum (Gibco) and 1 µg/ml puromycin (Sigma-Aldrich). To determine potency, SH-SY5Y AP-1 cells were seeded into 96-well plates (20,000 cells/well) and incubated overnight. After stimulation with MNC^aposec^ or its purified components, luciferase substrate (SteadyGlo; Promega) was added to all wells and plates were read in a luminescence reader (EnVision, Perkin Elmer or Centro LB960, Berthold).

### HSP27 phosphorylation

To detect HSP27 phosphorylation at residue Ser82, A549 cells were treated for 30 minutes, fixed, and permeabilized. Phosphorylation was assessed by sequential addition of an antibody detecting phosphorylated HSP27, followed by a peroxidase-conjugated anti-rabbit IgG antibody and chemiluminescent peroxidase substrate. The plates were read in the luminescence reader (EnVision, Perkin Elmer or Centro LB960, Berthold). The relative potency was calculated for the analyzed samples compared to the standard using the PLA software (Stegmann Systems PLA).

### Reverse transcription(RT)-quantitative polymerase chain reaction (qPCR)

Cultured aortic rings were manually excised together with outgrown cells from the surrounding fibrin matrix. RNA isolation, RT and qPCR were performed as described previously^80^.

### qPCR probes

Murine primers and probes were obtained from Thermo Fisher Scientific. Probes were conjugated to 5′ 6-carboxyfluorescein-major groove binder (FAM-MGB) and 3′ non-fluorescent quencher (NFQ). Assay IDs were: *Gapdh* Mm99999915_g1; *Vegfa* Mm00437306_m1; *Ena-78/Cxcl5* Mm00436451_g1.

### Statistical analysis

Data were statistically evaluated using GraphPad Prism 6 software (GraphPad Software Inc., LA Jolla, CA, US). For statistical analysis of planimetric data, repeated measures two-way ANOVA for matched subjects (time course data) was performed. Bonferroni post-tests were carried out to compare test groups versus controls. Data obtained by aortic ring assays were evaluated by two-way ANOVA and Sidak and Dunnett multiple comparison *post hoc* tests.

## Supplementary information

Supplementary Figures
