# Supplementary material for: Different pro-angiogenic potential of γ-irradiated PBMC-derived secretome and its subfractions
Source: Sci Rep. 2018 Dec 20;8:18016. doi: 10.1038/s41598-018-36928-6 (PMC6301954; doi:10.1038/s41598-018-36928-6)
Supplement: Supplementary file 1 — Supplementary Figures [file 41598_2018_36928_MOESM1_ESM.docx]

**Supplementary Information**

**Different pro-angiogenic potential of γ-irradiated PBMC-derived secretome and its subfractions**

Tanja Wagner, Denise Traxler, Elisabeth Simader, Lucian Beer, Marie-Sophie Narzt, Florian Gruber, Sibylle Madlener, Maria Laggner, Michael Erb, Vera Vorstandlechner, Alfred Gugerell, Christine Radtke, Massimiliano Gnecchi, Anja Peterbauer, Maria Gschwandtner, Erwin Tschachler, Claudia Keibl, Paul Slezak, Hendrik J. Ankersmit, and Michael Mildner

**Supplementary figure S1**

**
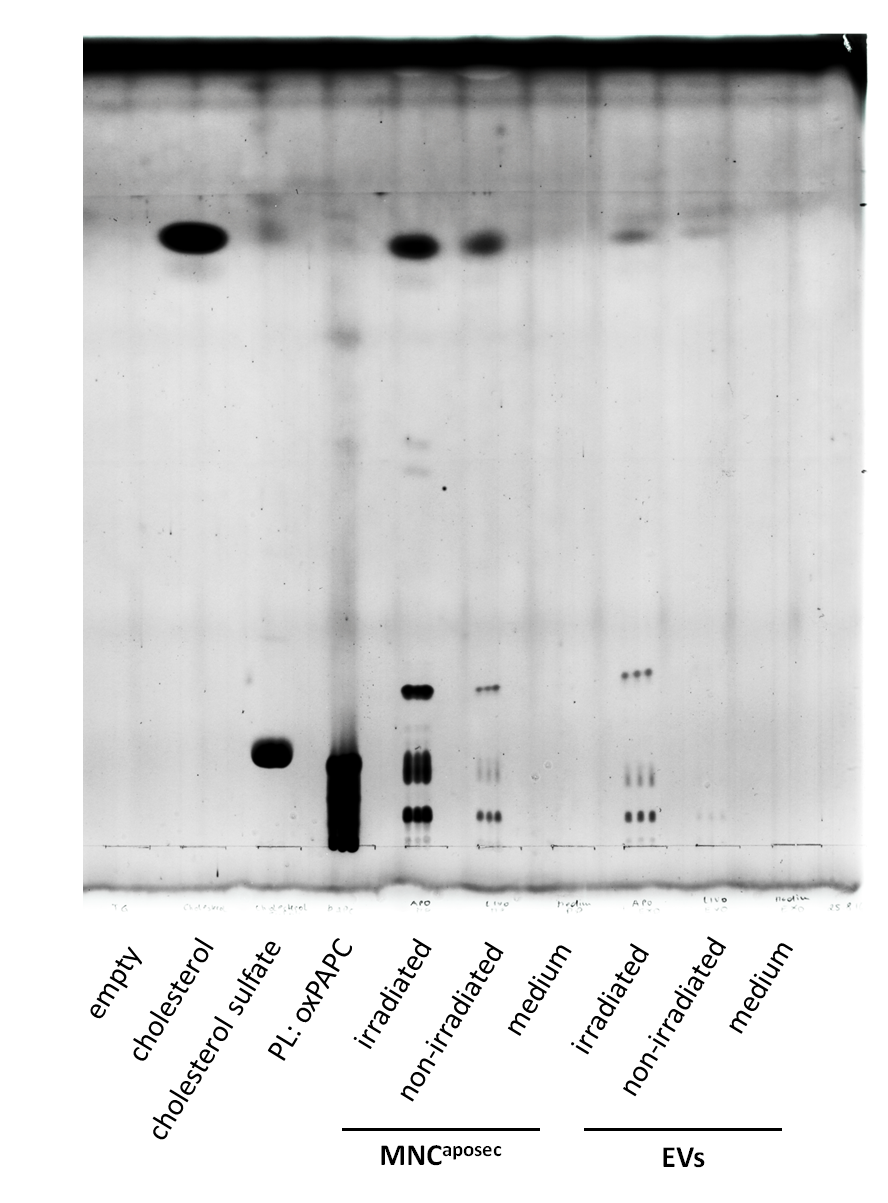
**

**Fig. S1. Full-length silica gel of TLC.** TLC of lipid species present in MNC^aposec^ and EVs. Cholesterol, cholesterol sulfate, and partially oxidized PAPC served as controls for polar lipids.

**Supplementary figure S2**


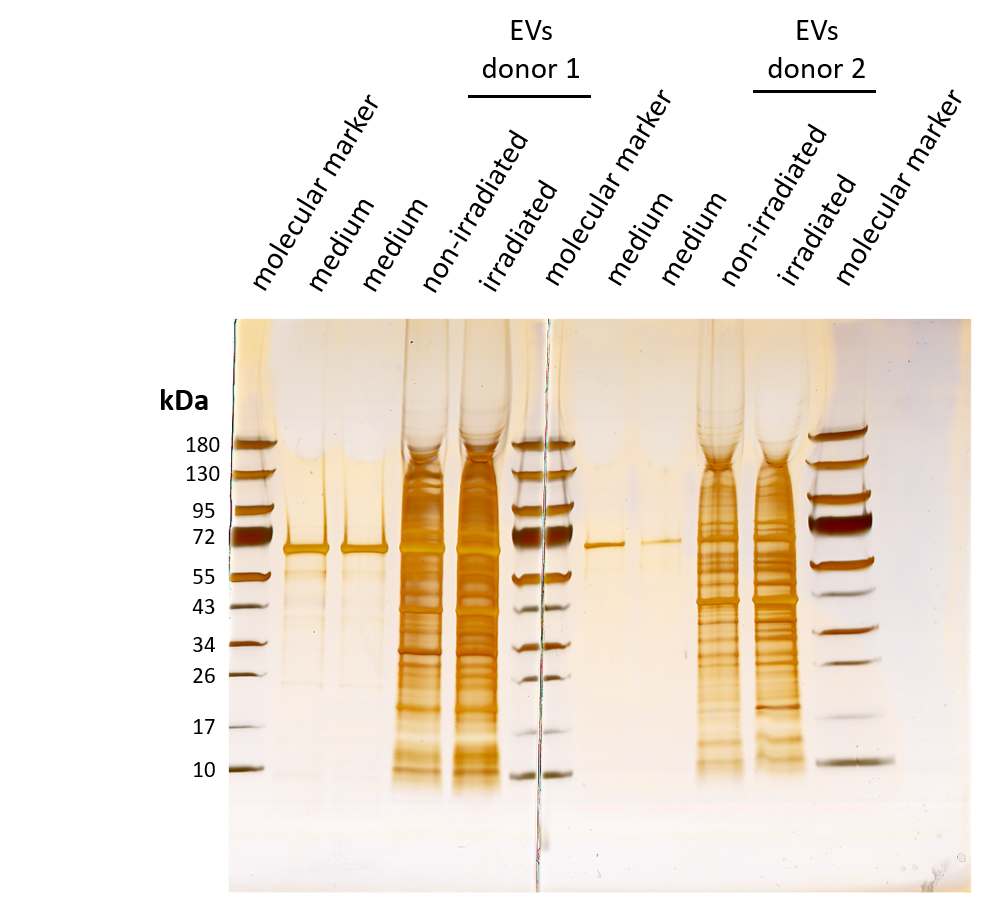
 **Fig. S2. Full-length gel of silver-stained proteins present in EVs.** Proteins released by irradiated and non-irradiated PBMC in EVs were separated by SDS-PAGE and quantitatively assessed by silver staining.

**Supplementary figure S3**


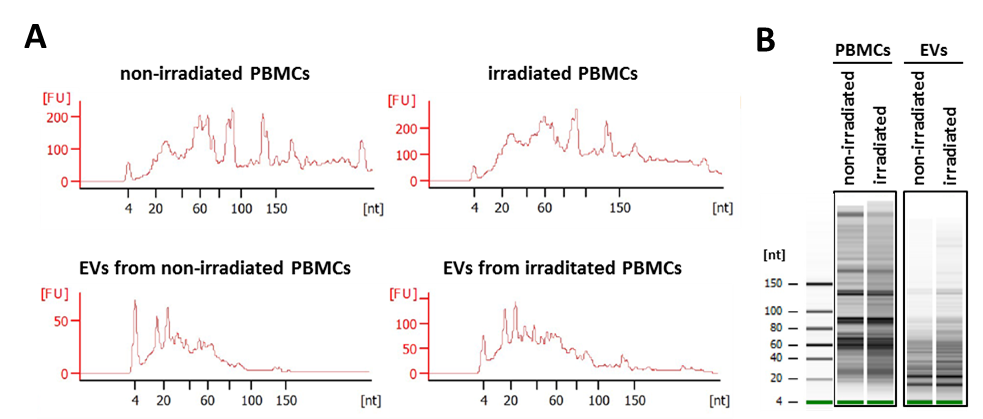


Fig. S3. RNA length profile for PBMCs and EVs. Size distribution profile of RNA fragments as determined by an Agilent Bioanalyzer. A pool of 10 donors was used for the analysis. (A) Electropherograms. (B) Gel images. PBMCs contained short and large RNA fragments. In contrast, PBMC-derived EVs contained mainly short RNA fragments of up to 80 nucleotides.

**Supplementary figure S4**


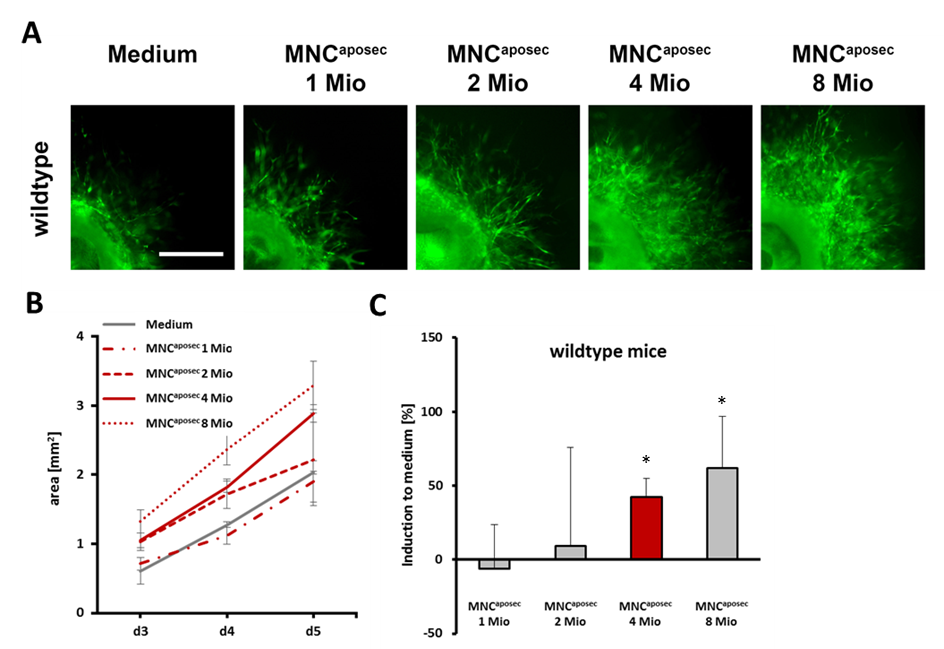


Fig. S4. Assessment of the angiogenic properties of MNC^aposec^ in a murine aortic ring assay. (A) Representative immunofluorescent images of aortic rings treated with different doses of MNC^aposec^ after 5 days cultivation. Scale bar = 500 µm. (B) The area of sprouted vessels on days 3, 4, and 5. (C) Induction of vessel sprouting after MNC^aposec^ treatment on day 5. Data are means +/- SD. Mean represents statistical analysis three donors; *p<0.05

**Supplementary figure S5**


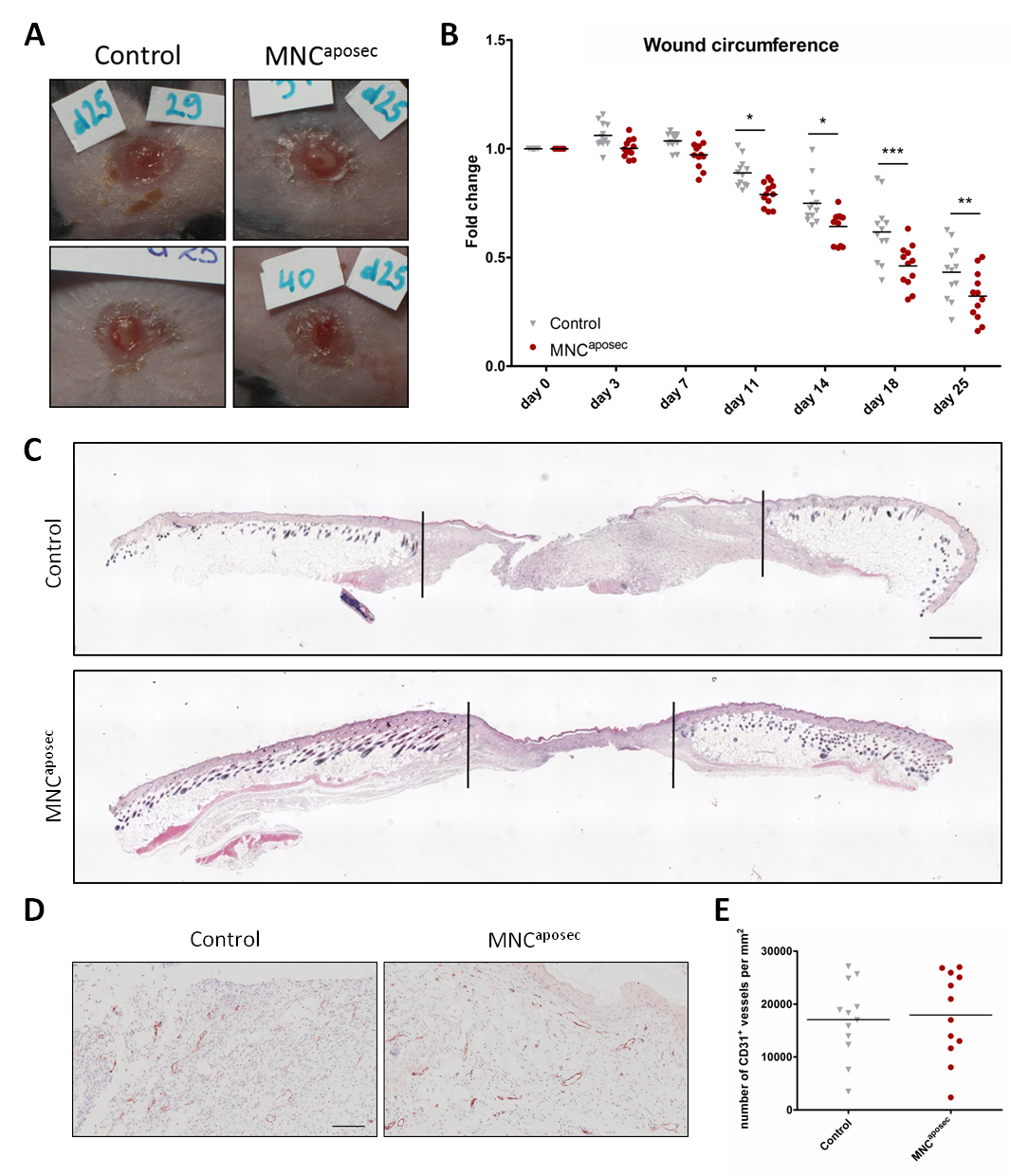


Fig. S5. MNC^aposec^ enhances wound healing in diabetic mice. (A) Representative photographs from wounds in diabetic mice (n=12 for each group) on day 25. The wounds were topically treated with 100 µl of therapeutic agent (MNC^aposec^; concentration of 25 × 10^6^ PBMCs/ml). Drug vehicle treatment served as a control. (B) Wound circumferences were measured on days 0 (initial wound), 3, 7, 11, 14, 18, and 25 after wounding. Starting from day 11 post-wounding, MNC^aposec^ significantly reduced the wound circumference. *p<0.05; **p<0.01; ***p<0.001. (C) H&E staining of a representative wound section from MNC^aposec^ and control-treated mice 25 days post-wounding. Vertical lines indicate the edges of the wound section. Scale bar = 1 mm. (D) Representative immunohistochemistry for CD31 in wounds on control and MNC^aposec^-treated diabetic mice 25 days post-wounding. Scale bar = 100 µm. (E) Quantitative analysis of CD31-positive cells in wounds for both groups. No significant difference in CD31 expression was found between the control and MNC^aposec^ groups at day 25.

**Supplementary figure S6**

**
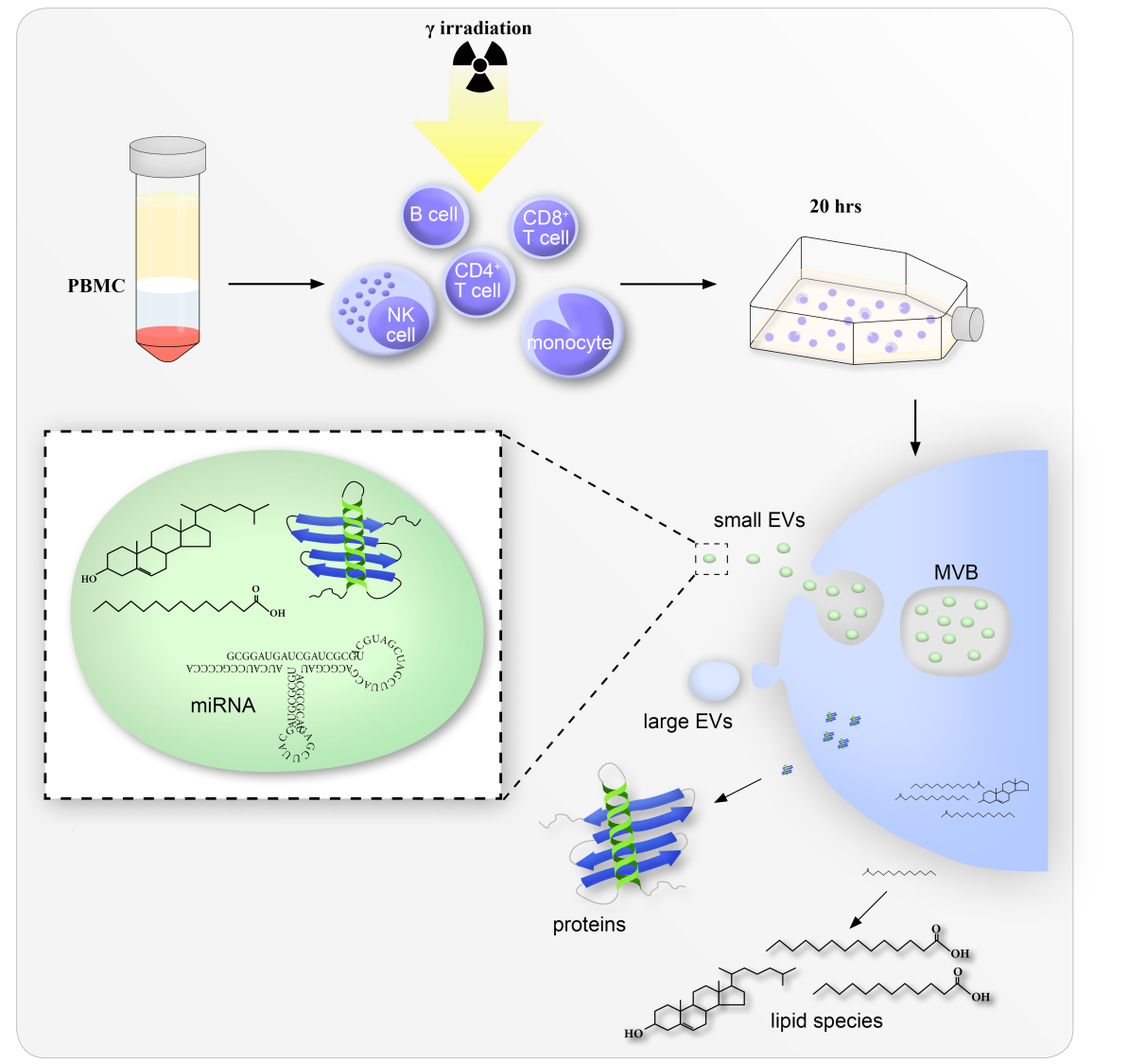
**

Fig. S6. Overview of MNC^aposec^ constituents. MNC^aposec^, the secretome of irradiated and cultured PBMCs, is composed of a variety of molecules, including EVs, proteins, and lipids. In turn, the EVs contain proteins, lipids, and miRNAs, which were investigated in more detail.

Table S1. List of proteins identified in EVs derived from irradiated and non-irradiated PBMCs.

| Accession | Protein Name |
| --- | --- |
| ALBU_HUMAN | Serum albumin OS=Homo sapiens GN=ALB PE=1 SV=2 |
| ACTG_HUMAN | Actin, cytoplasmic 2 OS=Homo sapiens GN=ACTG1 PE=1 SV=1 |
| ANXA6_HUMAN | Annexin A6 OS=Homo sapiens GN=ANXA6 PE=1 SV=3 |
| FLNA_HUMAN | Filamin-A OS=Homo sapiens GN=FLNA PE=1 SV=4 |
| PTPRC_HUMAN | Receptor-type tyrosine-protein phosphatase C OS=Homo sapiens GN=PTPRC PE=1 SV=2 |
| PLSL_HUMAN | Plastin-2 OS=Homo sapiens GN=LCP1 PE=1 SV=6 |
| TLN1_HUMAN | Talin-1 OS=Homo sapiens GN=TLN1 PE=1 SV=3 |
| MOES_HUMAN | Moesin OS=Homo sapiens GN=MSN PE=1 SV=3 |
| ANXA2_HUMAN | Annexin A2 OS=Homo sapiens GN=ANXA2 PE=1 SV=2 |
| ITA2B_HUMAN | Integrin alpha-IIb OS=Homo sapiens GN=ITGA2B PE=1 SV=3 |
| ENOA_HUMAN | Alpha-enolase OS=Homo sapiens GN=ENO1 PE=1 SV=2 |
| PERM_HUMAN | Myeloperoxidase OS=Homo sapiens GN=MPO PE=1 SV=1 |
| ITB2_HUMAN | Integrin beta-2 OS=Homo sapiens GN=ITGB2 PE=1 SV=2 |
| TSP1_HUMAN | Thrombospondin-1 OS=Homo sapiens GN=THBS1 PE=1 SV=2 |
| KPYM_HUMAN | Pyruvate kinase PKM OS=Homo sapiens GN=PKM PE=1 SV=4 |
| ANXA1_HUMAN | Annexin A1 OS=Homo sapiens GN=ANXA1 PE=1 SV=2 |
| ITB3_HUMAN | Integrin beta-3 OS=Homo sapiens GN=ITGB3 PE=1 SV=2 |
| H4_HUMAN | Histone H4 OS=Homo sapiens GN=HIST1H4A PE=1 SV=2 |
| ANXA5_HUMAN | Annexin A5 OS=Homo sapiens GN=ANXA5 PE=1 SV=2 |
| TBB4B_HUMAN | Tubulin beta-4B chain OS=Homo sapiens GN=TUBB4B PE=1 SV=1 |
| S10A9_HUMAN | Protein S100-A9 OS=Homo sapiens GN=S100A9 PE=1 SV=1 |
| ITAM_HUMAN | Integrin alpha-M OS=Homo sapiens GN=ITGAM PE=1 SV=2 |
| GNAI2_HUMAN | Guanine nucleotide-binding protein G(i) subunit alpha-2 OS=Homo sapiens GN=GNAI2 PE=1 SV=3 |
| G3P_HUMAN | Glyceraldehyde-3-phosphate dehydrogenase OS=Homo sapiens GN=GAPDH PE=1 SV=3 |
| COF1_HUMAN | Cofilin-1 OS=Homo sapiens GN=CFL1 PE=1 SV=3 |
| S10A8_HUMAN | Protein S100-A8 OS=Homo sapiens GN=S100A8 PE=1 SV=1 |
| CLH1_HUMAN | Clathrin heavy chain 1 OS=Homo sapiens GN=CLTC PE=1 SV=5 |
| ACTN1_HUMAN | Alpha-actinin-1 OS=Homo sapiens GN=ACTN1 PE=1 SV=2 |
| IQGA1_HUMAN | Ras GTPase-activating-like protein IQGAP1 OS=Homo sapiens GN=IQGAP1 PE=1 SV=1 |
| EF1A3_HUMAN | Putative elongation factor 1-alpha-like 3 OS=Homo sapiens GN=EEF1A1P5 PE=5 SV=1 |
| PROF1_HUMAN | Profilin-1 OS=Homo sapiens GN=PFN1 PE=1 SV=2 |
| PPIA_HUMAN | Peptidyl-prolyl cis-trans isomerase A OS=Homo sapiens GN=PPIA PE=1 SV=2 |
| HSP7C_HUMAN | Heat shock cognate 71 kDa protein OS=Homo sapiens GN=HSPA8 PE=1 SV=1 |
| ALDOA_HUMAN | Fructose-bisphosphate aldolase A OS=Homo sapiens GN=ALDOA PE=1 SV=2 |
| 1A02_HUMAN | HLA class I histocompatibility antigen, A-2 alpha chain OS=Homo sapiens GN=HLA-A PE=1 SV=1 |
| TBA4A_HUMAN | Tubulin alpha-4A chain OS=Homo sapiens GN=TUBA4A PE=1 SV=1 |
| HS90A_HUMAN | Heat shock protein HSP 90-alpha OS=Homo sapiens GN=HSP90AA1 PE=1 SV=5 |
| 1433Z_HUMAN | 14-3-3 protein zeta/delta OS=Homo sapiens GN=YWHAZ PE=1 SV=1 |
| RAP1B_HUMAN | Ras-related protein Rap-1b OS=Homo sapiens GN=RAP1B PE=1 SV=1 |
| HBB_HUMAN | Hemoglobin subunit beta OS=Homo sapiens GN=HBB PE=1 SV=2 |
| H2B1C_HUMAN | Histone H2B type 1-C/E/F/G/I OS=Homo sapiens GN=HIST1H2BC PE=1 SV=4 |
| COR1A_HUMAN | Coronin-1A OS=Homo sapiens GN=CORO1A PE=1 SV=4 |
| GRP78_HUMAN | 78 kDa glucose-regulated protein OS=Homo sapiens GN=HSPA5 PE=1 SV=2 |
| MVP_HUMAN | Major vault protein OS=Homo sapiens GN=MVP PE=1 SV=4 |
| PDC6I_HUMAN | Programmed cell death 6-interacting protein OS=Homo sapiens GN=PDCD6IP PE=1 SV=1 |
| ITAL_HUMAN | Integrin alpha-L OS=Homo sapiens GN=ITGAL PE=1 SV=3 |
| URP2_HUMAN | Fermitin family homolog 3 OS=Homo sapiens GN=FERMT3 PE=1 SV=1 |
| CAP1_HUMAN | Adenylyl cyclase-associated protein 1 OS=Homo sapiens GN=CAP1 PE=1 SV=5 |
| CLIC1_HUMAN | Chloride intracellular channel protein 1 OS=Homo sapiens GN=CLIC1 PE=1 SV=4 |
| AT1A1_HUMAN | Sodium/potassium-transporting ATPase subunit alpha-1 OS=Homo sapiens GN=ATP1A1 PE=1 SV=1 |
| VINC_HUMAN | Vinculin OS=Homo sapiens GN=VCL PE=1 SV=4 |
| ARP3_HUMAN | Actin-related protein 3 OS=Homo sapiens GN=ACTR3 PE=1 SV=3 |
| ENPL_HUMAN | Endoplasmin OS=Homo sapiens GN=HSP90B1 PE=1 SV=1 |
| 2B17_HUMAN | HLA class II histocompatibility antigen, DRB1-7 beta chain OS=Homo sapiens GN=HLA-DRB1 PE=1 SV=1 |
| RAC2_HUMAN | Ras-related C3 botulinum toxin substrate 2 OS=Homo sapiens GN=RAC2 PE=1 SV=1 |
| TKT_HUMAN | Transketolase OS=Homo sapiens GN=TKT PE=1 SV=3 |
| ANX11_HUMAN | Annexin A11 OS=Homo sapiens GN=ANXA11 PE=1 SV=1 |
| CDC42_HUMAN | Cell division control protein 42 homolog OS=Homo sapiens GN=CDC42 PE=1 SV=2 |
| GBB2_HUMAN | Guanine nucleotide-binding protein G(I)/G(S)/G(T) subunit beta-2 OS=Homo sapiens GN=GNB2 PE=1 SV=3 |
| PDIA3_HUMAN | Protein disulfide-isomerase A3 OS=Homo sapiens GN=PDIA3 PE=1 SV=4 |
| CD36_HUMAN | Platelet glycoprotein 4 OS=Homo sapiens GN=CD36 PE=1 SV=2 |
| RHOA_HUMAN | Transforming protein RhoA OS=Homo sapiens GN=RHOA PE=1 SV=1 |
| CD44_HUMAN | CD44 antigen OS=Homo sapiens GN=CD44 PE=1 SV=3 |
| UBB_HUMAN | Polyubiquitin-B OS=Homo sapiens GN=UBB PE=1 SV=1 |
| ITB1_HUMAN | Integrin beta-1 OS=Homo sapiens GN=ITGB1 PE=1 SV=2 |
| STOM_HUMAN | Erythrocyte band 7 integral membrane protein OS=Homo sapiens GN=STOM PE=1 SV=3 |
| HS90B_HUMAN | Heat shock protein HSP 90-beta OS=Homo sapiens GN=HSP90AB1 PE=1 SV=4 |
| PECA1_HUMAN | Platelet endothelial cell adhesion molecule OS=Homo sapiens GN=PECAM1 PE=1 SV=1 |
| ARF1_HUMAN | ADP-ribosylation factor 1 OS=Homo sapiens GN=ARF1 PE=1 SV=2 |
| ELNE_HUMAN | Neutrophil elastase OS=Homo sapiens GN=ELANE PE=1 SV=1 |
| LDHA_HUMAN | L-lactate dehydrogenase A chain OS=Homo sapiens GN=LDHA PE=1 SV=2 |
| ANXA4_HUMAN | Annexin A4 OS=Homo sapiens GN=ANXA4 PE=1 SV=4 |
| DRA_HUMAN | HLA class II histocompatibility antigen, DR alpha chain OS=Homo sapiens GN=HLA-DRA PE=1 SV=1 |
| 1B07_HUMAN | HLA class I histocompatibility antigen, B-7 alpha chain OS=Homo sapiens GN=HLA-B PE=1 SV=3 |
| MYO1G_HUMAN | Unconventional myosin-Ig OS=Homo sapiens GN=MYO1G PE=1 SV=2 |
| PRDX1_HUMAN | Peroxiredoxin-1 OS=Homo sapiens GN=PRDX1 PE=1 SV=1 |
| B2MG_HUMAN | Beta-2-microglobulin OS=Homo sapiens GN=B2M PE=1 SV=1 |
| ICAM3_HUMAN | Intercellular adhesion molecule 3 OS=Homo sapiens GN=ICAM3 PE=1 SV=2 |
| HS71A_HUMAN | Heat shock 70 kDa protein 1A OS=Homo sapiens GN=HSPA1A PE=1 SV=1 |
| F13A_HUMAN | Coagulation factor XIII A chain OS=Homo sapiens GN=F13A1 PE=1 SV=4 |
| GBB1_HUMAN | Guanine nucleotide-binding protein G(I)/G(S)/G(T) subunit beta-1 OS=Homo sapiens GN=GNB1 PE=1 SV=3 |
| IGHM_HUMAN | Immunoglobulin heavy constant mu OS=Homo sapiens GN=IGHM PE=1 SV=4 |
| 6PGD_HUMAN | 6-phosphogluconate dehydrogenase, decarboxylating OS=Homo sapiens GN=PGD PE=1 SV=3 |
| TBA1A_HUMAN | Tubulin alpha-1A chain OS=Homo sapiens GN=TUBA1A PE=1 SV=1 |
| RHOG_HUMAN | Rho-related GTP-binding protein RhoG OS=Homo sapiens GN=RHOG PE=1 SV=1 |
| ITAX_HUMAN | Integrin alpha-X OS=Homo sapiens GN=ITGAX PE=1 SV=3 |
| RAB7A_HUMAN | Ras-related protein Rab-7a OS=Homo sapiens GN=RAB7A PE=1 SV=1 |
| RB11B_HUMAN | Ras-related protein Rab-11B OS=Homo sapiens GN=RAB11B PE=1 SV=4 |
| PTPRJ_HUMAN | Receptor-type tyrosine-protein phosphatase eta OS=Homo sapiens GN=PTPRJ PE=1 SV=3 |
| ANXA7_HUMAN | Annexin A7 OS=Homo sapiens GN=ANXA7 PE=1 SV=3 |
| PTN6_HUMAN | Tyrosine-protein phosphatase non-receptor type 6 OS=Homo sapiens GN=PTPN6 PE=1 SV=1 |
| GAPR1_HUMAN | Golgi-associated plant pathogenesis-related protein 1 OS=Homo sapiens GN=GLIPR2 PE=1 SV=3 |
| NHRF1_HUMAN | Na(+)/H(+) exchange regulatory cofactor NHE-RF1 OS=Homo sapiens GN=SLC9A3R1 PE=1 SV=4 |
| ADA10_HUMAN | Disintegrin and metalloproteinase domain-containing protein 10 OS=Homo sapiens GN=ADAM10 PE=1 SV=1 |
| RS3_HUMAN | 40S ribosomal protein S3 OS=Homo sapiens GN=RPS3 PE=1 SV=2 |
| RSU1_HUMAN | Ras suppressor protein 1 OS=Homo sapiens GN=RSU1 PE=1 SV=3 |
| RAN_HUMAN | GTP-binding nuclear protein Ran OS=Homo sapiens GN=RAN PE=1 SV=3 |
| LYN_HUMAN | Tyrosine-protein kinase Lyn OS=Homo sapiens GN=LYN PE=1 SV=3 |
| SAMH1_HUMAN | Deoxynucleoside triphosphate triphosphohydrolase SAMHD1 OS=Homo sapiens GN=SAMHD1 PE=1 SV=2 |
| TBB1_HUMAN | Tubulin beta-1 chain OS=Homo sapiens GN=TUBB1 PE=1 SV=1 |
| GPIX_HUMAN | Platelet glycoprotein IX OS=Homo sapiens GN=GP9 PE=1 SV=3 |
| FLOT1_HUMAN | Flotillin-1 OS=Homo sapiens GN=FLOT1 PE=1 SV=3 |
| IGKC_HUMAN | Immunoglobulin kappa constant OS=Homo sapiens GN=IGKC PE=1 SV=2 |
| CTL2_HUMAN | Choline transporter-like protein 2 OS=Homo sapiens GN=SLC44A2 PE=1 SV=3 |
| STX7_HUMAN | Syntaxin-7 OS=Homo sapiens GN=STX7 PE=1 SV=4 |

Table S2. List of 377 proteins identified exclusively in EVs derived from irradiated PBMCs.

| Accession | Protein Name |
| --- | --- |
| VIME_HUMAN | Vimentin OS=Homo sapiens GN=VIM PE=1 SV=4 |
| PDIA1_HUMAN | Protein disulfide-isomerase OS=Homo sapiens GN=P4HB PE=1 SV=3 |
| AHNK_HUMAN | Neuroblast differentiation-associated protein AHNAK OS=Homo sapiens GN=AHNAK PE=1 SV=2 |
| PGK1_HUMAN | Phosphoglycerate kinase 1 OS=Homo sapiens GN=PGK1 PE=1 SV=3 |
| EF2_HUMAN | Elongation factor 2 OS=Homo sapiens GN=EEF2 PE=1 SV=4 |
| PLEC_HUMAN | Plectin OS=Homo sapiens GN=PLEC PE=1 SV=3 |
| TPIS_HUMAN | Triosephosphate isomerase OS=Homo sapiens GN=TPI1 PE=1 SV=3 |
| WDR1_HUMAN | WD repeat-containing protein 1 OS=Homo sapiens GN=WDR1 PE=1 SV=4 |
| ACTN4_HUMAN | Alpha-actinin-4 OS=Homo sapiens GN=ACTN4 PE=1 SV=2 |
| GELS_HUMAN | Gelsolin OS=Homo sapiens GN=GSN PE=1 SV=1 |
| ATPB_HUMAN | ATP synthase subunit beta, mitochondrial OS=Homo sapiens GN=ATP5B PE=1 SV=3 |
| GDIR2_HUMAN | Rho GDP-dissociation inhibitor 2 OS=Homo sapiens GN=ARHGDIB PE=1 SV=3 |
| GDIB_HUMAN | Rab GDP dissociation inhibitor beta OS=Homo sapiens GN=GDI2 PE=1 SV=2 |
| COTL1_HUMAN | Coactosin-like protein OS=Homo sapiens GN=COTL1 PE=1 SV=3 |
| ROA2_HUMAN | Heterogeneous nuclear ribonucleoproteins A2/B1 OS=Homo sapiens GN=HNRNPA2B1 PE=1 SV=2 |
| CALR_HUMAN | Calreticulin OS=Homo sapiens GN=CALR PE=1 SV=1 |
| ATPA_HUMAN | ATP synthase subunit alpha, mitochondrial OS=Homo sapiens GN=ATP5A1 PE=1 SV=1 |
| TERA_HUMAN | Transitional endoplasmic reticulum ATPase OS=Homo sapiens GN=VCP PE=1 SV=4 |
| TAGL2_HUMAN | Transgelin-2 OS=Homo sapiens GN=TAGLN2 PE=1 SV=3 |
| ARPC2_HUMAN | Actin-related protein 2/3 complex subunit 2 OS=Homo sapiens GN=ARPC2 PE=1 SV=1 |
| EZRI_HUMAN | Ezrin OS=Homo sapiens GN=EZR PE=1 SV=4 |
| ARP2_HUMAN | Actin-related protein 2 OS=Homo sapiens GN=ACTR2 PE=1 SV=1 |
| AMPN_HUMAN | Aminopeptidase N OS=Homo sapiens GN=ANPEP PE=1 SV=4 |
| PGAM1_HUMAN | Phosphoglycerate mutase 1 OS=Homo sapiens GN=PGAM1 PE=1 SV=2 |
| LEG1_HUMAN | Galectin-1 OS=Homo sapiens GN=LGALS1 PE=1 SV=2 |
| CH60_HUMAN | 60 kDa heat shock protein, mitochondrial OS=Homo sapiens GN=HSPD1 PE=1 SV=2 |
| HNRPK_HUMAN | Heterogeneous nuclear ribonucleoprotein K OS=Homo sapiens GN=HNRNPK PE=1 SV=1 |
| ARC1B_HUMAN | Actin-related protein 2/3 complex subunit 1B OS=Homo sapiens GN=ARPC1B PE=1 SV=3 |
| LDHB_HUMAN | L-lactate dehydrogenase B chain OS=Homo sapiens GN=LDHB PE=1 SV=2 |
| CAPZB_HUMAN | F-actin-capping protein subunit beta OS=Homo sapiens GN=CAPZB PE=1 SV=4 |
| ARPC4_HUMAN | Actin-related protein 2/3 complex subunit 4 OS=Homo sapiens GN=ARPC4 PE=1 SV=3 |
| GP1BB_HUMAN | Platelet glycoprotein Ib beta chain OS=Homo sapiens GN=GP1BB PE=1 SV=1 |
| LKHA4_HUMAN | Leukotriene A-4 hydrolase OS=Homo sapiens GN=LTA4H PE=1 SV=2 |
| FIBG_HUMAN | Fibrinogen gamma chain OS=Homo sapiens GN=FGG PE=1 SV=3 |
| LYSC_HUMAN | Lysozyme C OS=Homo sapiens GN=LYZ PE=1 SV=1 |
| UBA1_HUMAN | Ubiquitin-like modifier-activating enzyme 1 OS=Homo sapiens GN=UBA1 PE=1 SV=3 |
| CATA_HUMAN | Catalase OS=Homo sapiens GN=CAT PE=1 SV=3 |
| CAZA1_HUMAN | F-actin-capping protein subunit alpha-1 OS=Homo sapiens GN=CAPZA1 PE=1 SV=3 |
| PSME1_HUMAN | Proteasome activator complex subunit 1 OS=Homo sapiens GN=PSME1 PE=1 SV=1 |
| PLF4_HUMAN | Platelet factor 4 OS=Homo sapiens GN=PF4 PE=1 SV=2 |
| ITA6_HUMAN | Integrin alpha-6 OS=Homo sapiens GN=ITGA6 PE=1 SV=5 |
| ILEU_HUMAN | Leukocyte elastase inhibitor OS=Homo sapiens GN=SERPINB1 PE=1 SV=1 |
| GSTP1_HUMAN | Glutathione S-transferase P OS=Homo sapiens GN=GSTP1 PE=1 SV=2 |
| PPIB_HUMAN | Peptidyl-prolyl cis-trans isomerase B OS=Homo sapiens GN=PPIB PE=1 SV=2 |
| MDHM_HUMAN | Malate dehydrogenase, mitochondrial OS=Homo sapiens GN=MDH2 PE=1 SV=3 |
| G6PI_HUMAN | Glucose-6-phosphate isomerase OS=Homo sapiens GN=GPI PE=1 SV=4 |
| TCPQ_HUMAN | T-complex protein 1 subunit theta OS=Homo sapiens GN=CCT8 PE=1 SV=4 |
| CXCL7_HUMAN | Platelet basic protein OS=Homo sapiens GN=PPBP PE=1 SV=3 |
| G6PD_HUMAN | Glucose-6-phosphate 1-dehydrogenase OS=Homo sapiens GN=G6PD PE=1 SV=4 |
| ITA4_HUMAN | Integrin alpha-4 OS=Homo sapiens GN=ITGA4 PE=1 SV=3 |
| TALDO_HUMAN | Transaldolase OS=Homo sapiens GN=TALDO1 PE=1 SV=2 |
| RL6_HUMAN | 60S ribosomal protein L6 OS=Homo sapiens GN=RPL6 PE=1 SV=3 |
| RACK1_HUMAN | Receptor of activated protein C kinase 1 OS=Homo sapiens GN=RACK1 PE=1 SV=3 |
| CYFP2_HUMAN | Cytoplasmic FMR1-interacting protein 2 OS=Homo sapiens GN=CYFIP2 PE=1 SV=2 |
| CY24B_HUMAN | Cytochrome b-245 heavy chain OS=Homo sapiens GN=CYBB PE=1 SV=2 |
| TCPA_HUMAN | T-complex protein 1 subunit alpha OS=Homo sapiens GN=TCP1 PE=1 SV=1 |
| FIBB_HUMAN | Fibrinogen beta chain OS=Homo sapiens GN=FGB PE=1 SV=2 |
| EHD1_HUMAN | EH domain-containing protein 1 OS=Homo sapiens GN=EHD1 PE=1 SV=2 |
| FIBA_HUMAN | Fibrinogen alpha chain OS=Homo sapiens GN=FGA PE=1 SV=2 |
| GANAB_HUMAN | Neutral alpha-glucosidase AB OS=Homo sapiens GN=GANAB PE=1 SV=3 |
| RLA0_HUMAN | 60S acidic ribosomal protein P0 OS=Homo sapiens GN=RPLP0 PE=1 SV=1 |
| CAN1_HUMAN | Calpain-1 catalytic subunit OS=Homo sapiens GN=CAPN1 PE=1 SV=1 |
| RAB1B_HUMAN | Ras-related protein Rab-1B OS=Homo sapiens GN=RAB1B PE=1 SV=1 |
| PARK7_HUMAN | Protein DJ-1 OS=Homo sapiens GN=PARK7 PE=1 SV=2 |
| ROA1_HUMAN | Heterogeneous nuclear ribonucleoprotein A1 OS=Homo sapiens GN=HNRNPA1 PE=1 SV=5 |
| NDKB_HUMAN | Nucleoside diphosphate kinase B OS=Homo sapiens GN=NME2 PE=1 SV=1 |
| 1433B_HUMAN | 14-3-3 protein beta/alpha OS=Homo sapiens GN=YWHAB PE=1 SV=3 |
| NUCL_HUMAN | Nucleolin OS=Homo sapiens GN=NCL PE=1 SV=3 |
| PNPH_HUMAN | Purine nucleoside phosphorylase OS=Homo sapiens GN=PNP PE=1 SV=2 |
| PSME2_HUMAN | Proteasome activator complex subunit 2 OS=Homo sapiens GN=PSME2 PE=1 SV=4 |
| EFHD2_HUMAN | EF-hand domain-containing protein D2 OS=Homo sapiens GN=EFHD2 PE=1 SV=1 |
| MDHC_HUMAN | Malate dehydrogenase, cytoplasmic OS=Homo sapiens GN=MDH1 PE=1 SV=4 |
| PRDX6_HUMAN | Peroxiredoxin-6 OS=Homo sapiens GN=PRDX6 PE=1 SV=3 |
| RPN1_HUMAN | Dolichyl-diphosphooligosaccharide--protein glycosyltransferase subunit 1 OS=Homo sapiens GN=RPN1 PE=1 SV=1 |
| TCPB_HUMAN | T-complex protein 1 subunit beta OS=Homo sapiens GN=CCT2 PE=1 SV=4 |
| 1433E_HUMAN | 14-3-3 protein epsilon OS=Homo sapiens GN=YWHAE PE=1 SV=1 |
| TCPG_HUMAN | T-complex protein 1 subunit gamma OS=Homo sapiens GN=CCT3 PE=1 SV=4 |
| ARPC3_HUMAN | Actin-related protein 2/3 complex subunit 3 OS=Homo sapiens GN=ARPC3 PE=1 SV=3 |
| RSSA_HUMAN | 40S ribosomal protein SA OS=Homo sapiens GN=RPSA PE=1 SV=4 |
| LRP1_HUMAN | Prolow-density lipoprotein receptor-related protein 1 OS=Homo sapiens GN=LRP1 PE=1 SV=2 |
| EF1G_HUMAN | Elongation factor 1-gamma OS=Homo sapiens GN=EEF1G PE=1 SV=3 |
| TYPH_HUMAN | Thymidine phosphorylase OS=Homo sapiens GN=TYMP PE=1 SV=2 |
| CAPG_HUMAN | Macrophage-capping protein OS=Homo sapiens GN=CAPG PE=1 SV=2 |
| ACLY_HUMAN | ATP-citrate synthase OS=Homo sapiens GN=ACLY PE=1 SV=3 |
| CAND1_HUMAN | Cullin-associated NEDD8-dissociated protein 1 OS=Homo sapiens GN=CAND1 PE=1 SV=2 |
| TCPE_HUMAN | T-complex protein 1 subunit epsilon OS=Homo sapiens GN=CCT5 PE=1 SV=1 |
| RS4X_HUMAN | 40S ribosomal protein S4, X isoform OS=Homo sapiens GN=RPS4X PE=1 SV=2 |
| ARPC5_HUMAN | Actin-related protein 2/3 complex subunit 5 OS=Homo sapiens GN=ARPC5 PE=1 SV=3 |
| TCPH_HUMAN | T-complex protein 1 subunit eta OS=Homo sapiens GN=CCT7 PE=1 SV=2 |
| AT2B4_HUMAN | Plasma membrane calcium-transporting ATPase 4 OS=Homo sapiens GN=ATP2B4 PE=1 SV=2 |
| 1B53_HUMAN | HLA class I histocompatibility antigen, B-53 alpha chain OS=Homo sapiens GN=HLA-B PE=1 SV=1 |
| FMNL1_HUMAN | Formin-like protein 1 OS=Homo sapiens GN=FMNL1 PE=1 SV=3 |
| GTR3_HUMAN | Solute carrier family 2, facilitated glucose transporter member 3 OS=Homo sapiens GN=SLC2A3 PE=1 SV=1 |
| OST48_HUMAN | Dolichyl-diphosphooligosaccharide--protein glycosyltransferase 48 kDa subunit OS=Homo sapiens GN=DDOST PE=1 SV=4 |
| CKAP4_HUMAN | Cytoskeleton-associated protein 4 OS=Homo sapiens GN=CKAP4 PE=1 SV=2 |
| TCPZ_HUMAN | T-complex protein 1 subunit zeta OS=Homo sapiens GN=CCT6A PE=1 SV=3 |
| SPB9_HUMAN | Serpin B9 OS=Homo sapiens GN=SERPINB9 PE=1 SV=1 |
| CATG_HUMAN | Cathepsin G OS=Homo sapiens GN=CTSG PE=1 SV=2 |
| PP1A_HUMAN | Serine/threonine-protein phosphatase PP1-alpha catalytic subunit OS=Homo sapiens GN=PPP1CA PE=1 SV=1 |
| DPYL2_HUMAN | Dihydropyrimidinase-related protein 2 OS=Homo sapiens GN=DPYSL2 PE=1 SV=1 |
| 4F2_HUMAN | 4F2 cell-surface antigen heavy chain OS=Homo sapiens GN=SLC3A2 PE=1 SV=3 |
| CPNS1_HUMAN | Calpain small subunit 1 OS=Homo sapiens GN=CAPNS1 PE=1 SV=1 |
| GRB2_HUMAN | Growth factor receptor-bound protein 2 OS=Homo sapiens GN=GRB2 PE=1 SV=1 |
| SPTB2_HUMAN | Spectrin beta chain, non-erythrocytic 1 OS=Homo sapiens GN=SPTBN1 PE=1 SV=2 |
| CH10_HUMAN | 10 kDa heat shock protein, mitochondrial OS=Homo sapiens GN=HSPE1 PE=1 SV=2 |
| LSP1_HUMAN | Lymphocyte-specific protein 1 OS=Homo sapiens GN=LSP1 PE=1 SV=1 |
| BST1_HUMAN | ADP-ribosyl cyclase/cyclic ADP-ribose hydrolase 2 OS=Homo sapiens GN=BST1 PE=1 SV=2 |
| PHB2_HUMAN | Prohibitin-2 OS=Homo sapiens GN=PHB2 PE=1 SV=2 |
| PHB_HUMAN | Prohibitin OS=Homo sapiens GN=PHB PE=1 SV=1 |
| IL16_HUMAN | Pro-interleukin-16 OS=Homo sapiens GN=IL16 PE=1 SV=4 |
| CD48_HUMAN | CD48 antigen OS=Homo sapiens GN=CD48 PE=1 SV=2 |
| VDAC1_HUMAN | Voltage-dependent anion-selective channel protein 1 OS=Homo sapiens GN=VDAC1 PE=1 SV=2 |
| MYO1F_HUMAN | Unconventional myosin-If OS=Homo sapiens GN=MYO1F PE=1 SV=3 |
| RINI_HUMAN | Ribonuclease inhibitor OS=Homo sapiens GN=RNH1 PE=1 SV=2 |
| GNAI3_HUMAN | Guanine nucleotide-binding protein G(k) subunit alpha OS=Homo sapiens GN=GNAI3 PE=1 SV=3 |
| KAD2_HUMAN | Adenylate kinase 2, mitochondrial OS=Homo sapiens GN=AK2 PE=1 SV=2 |
| GP1BA_HUMAN | Platelet glycoprotein Ib alpha chain OS=Homo sapiens GN=GP1BA PE=1 SV=2 |
| RS2_HUMAN | 40S ribosomal protein S2 OS=Homo sapiens GN=RPS2 PE=1 SV=2 |
| TCPD_HUMAN | T-complex protein 1 subunit delta OS=Homo sapiens GN=CCT4 PE=1 SV=4 |
| ADT2_HUMAN | ADP/ATP translocase 2 OS=Homo sapiens GN=SLC25A5 PE=1 SV=7 |
| HXK1_HUMAN | Hexokinase-1 OS=Homo sapiens GN=HK1 PE=1 SV=3 |
| IF5A1_HUMAN | Eukaryotic translation initiation factor 5A-1 OS=Homo sapiens GN=EIF5A PE=1 SV=2 |
| PEBP1_HUMAN | Phosphatidylethanolamine-binding protein 1 OS=Homo sapiens GN=PEBP1 PE=1 SV=3 |
| NIBAN_HUMAN | Protein Niban OS=Homo sapiens GN=FAM129A PE=1 SV=1 |
| CD97_HUMAN | CD97 antigen OS=Homo sapiens GN=CD97 PE=1 SV=4 |
| COR1C_HUMAN | Coronin-1C OS=Homo sapiens GN=CORO1C PE=1 SV=1 |
| HCLS1_HUMAN | Hematopoietic lineage cell-specific protein OS=Homo sapiens GN=HCLS1 PE=1 SV=3 |
| FLOT2_HUMAN | Flotillin-2 OS=Homo sapiens GN=FLOT2 PE=1 SV=2 |
| 2B13_HUMAN | HLA class II histocompatibility antigen, DRB1-3 chain OS=Homo sapiens GN=HLA-DRB1 PE=1 SV=2 |
| 1A03_HUMAN | HLA class I histocompatibility antigen, A-3 alpha chain OS=Homo sapiens GN=HLA-A PE=1 SV=2 |
| TPP2_HUMAN | Tripeptidyl-peptidase 2 OS=Homo sapiens GN=TPP2 PE=1 SV=4 |
| GSTO1_HUMAN | Glutathione S-transferase omega-1 OS=Homo sapiens GN=GSTO1 PE=1 SV=2 |
| SEPT7_HUMAN | Septin-7 OS=Homo sapiens GN=SEPT7 PE=1 SV=2 |
| SPTN1_HUMAN | Spectrin alpha chain, non-erythrocytic 1 OS=Homo sapiens GN=SPTAN1 PE=1 SV=3 |
| NCKPL_HUMAN | Nck-associated protein 1-like OS=Homo sapiens GN=NCKAP1L PE=1 SV=3 |
| PDIA6_HUMAN | Protein disulfide-isomerase A6 OS=Homo sapiens GN=PDIA6 PE=1 SV=1 |
| PSA7_HUMAN | Proteasome subunit alpha type-7 OS=Homo sapiens GN=PSMA7 PE=1 SV=1 |
| DHE3_HUMAN | Glutamate dehydrogenase 1, mitochondrial OS=Homo sapiens GN=GLUD1 PE=1 SV=2 |
| PGM1_HUMAN | Phosphoglucomutase-1 OS=Homo sapiens GN=PGM1 PE=1 SV=3 |
| QCR1_HUMAN | Cytochrome b-c1 complex subunit 1, mitochondrial OS=Homo sapiens GN=UQCRC1 PE=1 SV=3 |
| RAB14_HUMAN | Ras-related protein Rab-14 OS=Homo sapiens GN=RAB14 PE=1 SV=4 |
| RAB8A_HUMAN | Ras-related protein Rab-8A OS=Homo sapiens GN=RAB8A PE=1 SV=1 |
| PLEK_HUMAN | Pleckstrin OS=Homo sapiens GN=PLEK PE=1 SV=3 |
| FA49B_HUMAN | Protein FAM49B OS=Homo sapiens GN=FAM49B PE=1 SV=1 |
| GDIR1_HUMAN | Rho GDP-dissociation inhibitor 1 OS=Homo sapiens GN=ARHGDIA PE=1 SV=3 |
| VASP_HUMAN | Vasodilator-stimulated phosphoprotein OS=Homo sapiens GN=VASP PE=1 SV=3 |
| EST1_HUMAN | Liver carboxylesterase 1 OS=Homo sapiens GN=CES1 PE=1 SV=2 |
| RL7_HUMAN | 60S ribosomal protein L7 OS=Homo sapiens GN=RPL7 PE=1 SV=1 |
| CNDP2_HUMAN | Cytosolic non-specific dipeptidase OS=Homo sapiens GN=CNDP2 PE=1 SV=2 |
| IQGA2_HUMAN | Ras GTPase-activating-like protein IQGAP2 OS=Homo sapiens GN=IQGAP2 PE=1 SV=4 |
| TWF2_HUMAN | Twinfilin-2 OS=Homo sapiens GN=TWF2 PE=1 SV=2 |
| AT1B3_HUMAN | Sodium/potassium-transporting ATPase subunit beta-3 OS=Homo sapiens GN=ATP1B3 PE=1 SV=1 |
| XRCC6_HUMAN | X-ray repair cross-complementing protein 6 OS=Homo sapiens GN=XRCC6 PE=1 SV=2 |
| HNRH1_HUMAN | Heterogeneous nuclear ribonucleoprotein H OS=Homo sapiens GN=HNRNPH1 PE=1 SV=4 |
| VDAC2_HUMAN | Voltage-dependent anion-selective channel protein 2 OS=Homo sapiens GN=VDAC2 PE=1 SV=2 |
| ALDR_HUMAN | Aldose reductase OS=Homo sapiens GN=AKR1B1 PE=1 SV=3 |
| DBNL_HUMAN | Drebrin-like protein OS=Homo sapiens GN=DBNL PE=1 SV=1 |
| AN32A_HUMAN | Acidic leucine-rich nuclear phosphoprotein 32 family member A OS=Homo sapiens GN=ANP32A PE=1 SV=1 |
| STIP1_HUMAN | Stress-induced-phosphoprotein 1 OS=Homo sapiens GN=STIP1 PE=1 SV=1 |
| PTBP1_HUMAN | Polypyrimidine tract-binding protein 1 OS=Homo sapiens GN=PTBP1 PE=1 SV=1 |
| NAGK_HUMAN | N-acetyl-D-glucosamine kinase OS=Homo sapiens GN=NAGK PE=1 SV=4 |
| PRDX5_HUMAN | Peroxiredoxin-5, mitochondrial OS=Homo sapiens GN=PRDX5 PE=1 SV=4 |
| CAN2_HUMAN | Calpain-2 catalytic subunit OS=Homo sapiens GN=CAPN2 PE=1 SV=6 |
| DYHC1_HUMAN | Cytoplasmic dynein 1 heavy chain 1 OS=Homo sapiens GN=DYNC1H1 PE=1 SV=5 |
| PSA1_HUMAN | Proteasome subunit alpha type-1 OS=Homo sapiens GN=PSMA1 PE=1 SV=1 |
| PCBP1_HUMAN | Poly(rC)-binding protein 1 OS=Homo sapiens GN=PCBP1 PE=1 SV=2 |
| CD38_HUMAN | ADP-ribosyl cyclase/cyclic ADP-ribose hydrolase 1 OS=Homo sapiens GN=CD38 PE=1 SV=2 |
| 1A24_HUMAN | HLA class I histocompatibility antigen, A-24 alpha chain OS=Homo sapiens GN=HLA-A PE=1 SV=2 |
| XRCC5_HUMAN | X-ray repair cross-complementing protein 5 OS=Homo sapiens GN=XRCC5 PE=1 SV=3 |
| PTCA_HUMAN | Protein tyrosine phosphatase receptor type C-associated protein OS=Homo sapiens GN=PTPRCAP PE=1 SV=1 |
| PRDX2_HUMAN | Peroxiredoxin-2 OS=Homo sapiens GN=PRDX2 PE=1 SV=5 |
| HXK3_HUMAN | Hexokinase-3 OS=Homo sapiens GN=HK3 PE=1 SV=2 |
| S10AB_HUMAN | Protein S100-A11 OS=Homo sapiens GN=S100A11 PE=1 SV=2 |
| PLXB2_HUMAN | Plexin-B2 OS=Homo sapiens GN=PLXNB2 PE=1 SV=3 |
| TRFL_HUMAN | Lactotransferrin OS=Homo sapiens GN=LTF PE=1 SV=6 |
| CD3Z_HUMAN | T-cell surface glycoprotein CD3 zeta chain OS=Homo sapiens GN=CD247 PE=1 SV=2 |
| UB2V1_HUMAN | Ubiquitin-conjugating enzyme E2 variant 1 OS=Homo sapiens GN=UBE2V1 PE=1 SV=2 |
| CD37_HUMAN | Leukocyte antigen CD37 OS=Homo sapiens GN=CD37 PE=1 SV=2 |
| VPS35_HUMAN | Vacuolar protein sorting-associated protein 35 OS=Homo sapiens GN=VPS35 PE=1 SV=2 |
| ERP29_HUMAN | Endoplasmic reticulum resident protein 29 OS=Homo sapiens GN=ERP29 PE=1 SV=4 |
| STK10_HUMAN | Serine/threonine-protein kinase 10 OS=Homo sapiens GN=STK10 PE=1 SV=1 |
| PSA_HUMAN | Puromycin-sensitive aminopeptidase OS=Homo sapiens GN=NPEPPS PE=1 SV=2 |
| SFPQ_HUMAN | Splicing factor, proline- and glutamine-rich OS=Homo sapiens GN=SFPQ PE=1 SV=2 |
| RS18_HUMAN | 40S ribosomal protein S18 OS=Homo sapiens GN=RPS18 PE=1 SV=3 |
| DIAP1_HUMAN | Protein diaphanous homolog 1 OS=Homo sapiens GN=DIAPH1 PE=1 SV=2 |
| APT_HUMAN | Adenine phosphoribosyltransferase OS=Homo sapiens GN=APRT PE=1 SV=2 |
| PSA6_HUMAN | Proteasome subunit alpha type-6 OS=Homo sapiens GN=PSMA6 PE=1 SV=1 |
| CD14_HUMAN | Monocyte differentiation antigen CD14 OS=Homo sapiens GN=CD14 PE=1 SV=2 |
| 1433T_HUMAN | 14-3-3 protein theta OS=Homo sapiens GN=YWHAQ PE=1 SV=1 |
| PDIA4_HUMAN | Protein disulfide-isomerase A4 OS=Homo sapiens GN=PDIA4 PE=1 SV=2 |
| SAHH_HUMAN | Adenosylhomocysteinase OS=Homo sapiens GN=AHCY PE=1 SV=4 |
| IMB1_HUMAN | Importin subunit beta-1 OS=Homo sapiens GN=KPNB1 PE=1 SV=2 |
| PRTN3_HUMAN | Myeloblastin OS=Homo sapiens GN=PRTN3 PE=1 SV=3 |
| AP1B1_HUMAN | AP-1 complex subunit beta-1 OS=Homo sapiens GN=AP1B1 PE=1 SV=2 |
| UGPA_HUMAN | UTP--glucose-1-phosphate uridylyltransferase OS=Homo sapiens GN=UGP2 PE=1 SV=5 |
| 1B44_HUMAN | HLA class I histocompatibility antigen, B-44 alpha chain OS=Homo sapiens GN=HLA-B PE=1 SV=1 |
| RB27B_HUMAN | Ras-related protein Rab-27B OS=Homo sapiens GN=RAB27B PE=1 SV=4 |
| STXB2_HUMAN | Syntaxin-binding protein 2 OS=Homo sapiens GN=STXBP2 PE=1 SV=2 |
| PUR9_HUMAN | Bifunctional purine biosynthesis protein PURH OS=Homo sapiens GN=ATIC PE=1 SV=3 |
| IDHC_HUMAN | Isocitrate dehydrogenase [NADP] cytoplasmic OS=Homo sapiens GN=IDH1 PE=1 SV=2 |
| LEUK_HUMAN | Leukosialin OS=Homo sapiens GN=SPN PE=1 SV=1 |
| IGG1_HUMAN | Immunoglobulin gamma-1 heavy chain OS=Homo sapiens PE=1 SV=1 |
| AT2A3_HUMAN | Sarcoplasmic/endoplasmic reticulum calcium ATPase 3 OS=Homo sapiens GN=ATP2A3 PE=1 SV=2 |
| B3AT_HUMAN | Band 3 anion transport protein OS=Homo sapiens GN=SLC4A1 PE=1 SV=3 |
| GSHR_HUMAN | Glutathione reductase, mitochondrial OS=Homo sapiens GN=GSR PE=1 SV=2 |
| ASC_HUMAN | Apoptosis-associated speck-like protein containing a CARD OS=Homo sapiens GN=PYCARD PE=1 SV=2 |
| 1433G_HUMAN | 14-3-3 protein gamma OS=Homo sapiens GN=YWHAG PE=1 SV=2 |
| CR1_HUMAN | Complement receptor type 1 OS=Homo sapiens GN=CR1 PE=1 SV=3 |
| COPA_HUMAN | Coatomer subunit alpha OS=Homo sapiens GN=COPA PE=1 SV=2 |
| CNN2_HUMAN | Calponin-2 OS=Homo sapiens GN=CNN2 PE=1 SV=4 |
| CAZA2_HUMAN | F-actin-capping protein subunit alpha-2 OS=Homo sapiens GN=CAPZA2 PE=1 SV=3 |
| JAM1_HUMAN | Junctional adhesion molecule A OS=Homo sapiens GN=F11R PE=1 SV=1 |
| RL18_HUMAN | 60S ribosomal protein L18 OS=Homo sapiens GN=RPL18 PE=1 SV=2 |
| CPNE1_HUMAN | Copine-1 OS=Homo sapiens GN=CPNE1 PE=1 SV=1 |
| CISY_HUMAN | Citrate synthase, mitochondrial OS=Homo sapiens GN=CS PE=1 SV=2 |
| HSP74_HUMAN | Heat shock 70 kDa protein 4 OS=Homo sapiens GN=HSPA4 PE=1 SV=4 |
| EIF3B_HUMAN | Eukaryotic translation initiation factor 3 subunit B OS=Homo sapiens GN=EIF3B PE=1 SV=3 |
| EIF3A_HUMAN | Eukaryotic translation initiation factor 3 subunit A OS=Homo sapiens GN=EIF3A PE=1 SV=1 |
| RAB8B_HUMAN | Ras-related protein Rab-8B OS=Homo sapiens GN=RAB8B PE=1 SV=2 |
| 6PGL_HUMAN | 6-phosphogluconolactonase OS=Homo sapiens GN=PGLS PE=1 SV=2 |
| CD20_HUMAN | B-lymphocyte antigen CD20 OS=Homo sapiens GN=MS4A1 PE=1 SV=1 |
| EF1D_HUMAN | Elongation factor 1-delta OS=Homo sapiens GN=EEF1D PE=1 SV=5 |
| RAB2A_HUMAN | Ras-related protein Rab-2A OS=Homo sapiens GN=RAB2A PE=1 SV=1 |
| PSB1_HUMAN | Proteasome subunit beta type-1 OS=Homo sapiens GN=PSMB1 PE=1 SV=2 |
| CATD_HUMAN | Cathepsin D OS=Homo sapiens GN=CTSD PE=1 SV=1 |
| RPN2_HUMAN | Dolichyl-diphosphooligosaccharide--protein glycosyltransferase subunit 2 OS=Homo sapiens GN=RPN2 PE=1 SV=3 |
| 1433F_HUMAN | 14-3-3 protein eta OS=Homo sapiens GN=YWHAH PE=1 SV=4 |
| KAP0_HUMAN | cAMP-dependent protein kinase type I-alpha regulatory subunit OS=Homo sapiens GN=PRKAR1A PE=1 SV=1 |
| FUBP2_HUMAN | Far upstream element-binding protein 2 OS=Homo sapiens GN=KHSRP PE=1 SV=4 |
| 1C06_HUMAN | HLA class I histocompatibility antigen, Cw-6 alpha chain OS=Homo sapiens GN=HLA-C PE=1 SV=2 |
| PSB2_HUMAN | Proteasome subunit beta type-2 OS=Homo sapiens GN=PSMB2 PE=1 SV=1 |
| DX39B_HUMAN | Spliceosome RNA helicase DDX39B OS=Homo sapiens GN=DDX39B PE=1 SV=1 |
| SYHC_HUMAN | Histidine--tRNA ligase, cytoplasmic OS=Homo sapiens GN=HARS PE=1 SV=2 |
| GLOD4_HUMAN | Glyoxalase domain-containing protein 4 OS=Homo sapiens GN=GLOD4 PE=1 SV=1 |
| ECH1_HUMAN | Delta(3,5)-Delta(2,4)-dienoyl-CoA isomerase, mitochondrial OS=Homo sapiens GN=ECH1 PE=1 SV=2 |
| CD5_HUMAN | T-cell surface glycoprotein CD5 OS=Homo sapiens GN=CD5 PE=1 SV=2 |
| IF4A2_HUMAN | Eukaryotic initiation factor 4A-II OS=Homo sapiens GN=EIF4A2 PE=1 SV=2 |
| ICAM1_HUMAN | Intercellular adhesion molecule 1 OS=Homo sapiens GN=ICAM1 PE=1 SV=2 |
| DDX17_HUMAN | Probable ATP-dependent RNA helicase DDX17 OS=Homo sapiens GN=DDX17 PE=1 SV=2 |
| QCR2_HUMAN | Cytochrome b-c1 complex subunit 2, mitochondrial OS=Homo sapiens GN=UQCRC2 PE=1 SV=3 |
| VATB2_HUMAN | V-type proton ATPase subunit B, brain isoform OS=Homo sapiens GN=ATP6V1B2 PE=1 SV=3 |
| UBE2N_HUMAN | Ubiquitin-conjugating enzyme E2 N OS=Homo sapiens GN=UBE2N PE=1 SV=1 |
| ALDOC_HUMAN | Fructose-bisphosphate aldolase C OS=Homo sapiens GN=ALDOC PE=1 SV=2 |
| ACPH_HUMAN | Acylamino-acid-releasing enzyme OS=Homo sapiens GN=APEH PE=1 SV=4 |
| PSA5_HUMAN | Proteasome subunit alpha type-5 OS=Homo sapiens GN=PSMA5 PE=1 SV=3 |
| AL9A1_HUMAN | 4-trimethylaminobutyraldehyde dehydrogenase OS=Homo sapiens GN=ALDH9A1 PE=1 SV=3 |
| GMFG_HUMAN | Glia maturation factor gamma OS=Homo sapiens GN=GMFG PE=1 SV=1 |
| SEPT6_HUMAN | Septin-6 OS=Homo sapiens GN=SEPT6 PE=1 SV=4 |
| VDAC3_HUMAN | Voltage-dependent anion-selective channel protein 3 OS=Homo sapiens GN=VDAC3 PE=1 SV=1 |
| IST1_HUMAN | IST1 homolog OS=Homo sapiens GN=IST1 PE=1 SV=1 |
| TXND5_HUMAN | Thioredoxin domain-containing protein 5 OS=Homo sapiens GN=TXNDC5 PE=1 SV=2 |
| C1TC_HUMAN | C-1-tetrahydrofolate synthase, cytoplasmic OS=Homo sapiens GN=MTHFD1 PE=1 SV=3 |
| PSA3_HUMAN | Proteasome subunit alpha type-3 OS=Homo sapiens GN=PSMA3 PE=1 SV=2 |
| FUBP1_HUMAN | Far upstream element-binding protein 1 OS=Homo sapiens GN=FUBP1 PE=1 SV=3 |
| SEPT2_HUMAN | Septin-2 OS=Homo sapiens GN=SEPT2 PE=1 SV=1 |
| ITA5_HUMAN | Integrin alpha-5 OS=Homo sapiens GN=ITGA5 PE=1 SV=2 |
| AMPL_HUMAN | Cytosol aminopeptidase OS=Homo sapiens GN=LAP3 PE=1 SV=3 |
| RAP2B_HUMAN | Ras-related protein Rap-2b OS=Homo sapiens GN=RAP2B PE=1 SV=1 |
| RS16_HUMAN | 40S ribosomal protein S16 OS=Homo sapiens GN=RPS16 PE=1 SV=2 |
| PSB8_HUMAN | Proteasome subunit beta type-8 OS=Homo sapiens GN=PSMB8 PE=1 SV=3 |
| ARF6_HUMAN | ADP-ribosylation factor 6 OS=Homo sapiens GN=ARF6 PE=1 SV=2 |
| VAT1_HUMAN | Synaptic vesicle membrane protein VAT-1 homolog OS=Homo sapiens GN=VAT1 PE=1 SV=2 |
| FUMH_HUMAN | Fumarate hydratase, mitochondrial OS=Homo sapiens GN=FH PE=1 SV=3 |
| SH3L1_HUMAN | SH3 domain-binding glutamic acid-rich-like protein OS=Homo sapiens GN=SH3BGRL PE=1 SV=1 |
| PUR6_HUMAN | Multifunctional protein ADE2 OS=Homo sapiens GN=PAICS PE=1 SV=3 |
| 2AAA_HUMAN | Serine/threonine-protein phosphatase 2A 65 kDa regulatory subunit A alpha isoform OS=Homo sapiens GN=PPP2R1A PE=1 SV=4 |
| ESYT1_HUMAN | Extended synaptotagmin-1 OS=Homo sapiens GN=ESYT1 PE=1 SV=1 |
| RAB3D_HUMAN | Ras-related protein Rab-3D OS=Homo sapiens GN=RAB3D PE=1 SV=1 |
| RS19_HUMAN | 40S ribosomal protein S19 OS=Homo sapiens GN=RPS19 PE=1 SV=2 |
| GNAS1_HUMAN | Guanine nucleotide-binding protein G(s) subunit alpha isoforms XLas OS=Homo sapiens GN=GNAS PE=1 SV=2 |
| OSTF1_HUMAN | Osteoclast-stimulating factor 1 OS=Homo sapiens GN=OSTF1 PE=1 SV=2 |
| F10A1_HUMAN | Hsc70-interacting protein OS=Homo sapiens GN=ST13 PE=1 SV=2 |
| RL12_HUMAN | 60S ribosomal protein L12 OS=Homo sapiens GN=RPL12 PE=1 SV=1 |
| EIF3C_HUMAN | Eukaryotic translation initiation factor 3 subunit C OS=Homo sapiens GN=EIF3C PE=1 SV=1 |
| HMHA1_HUMAN | Rho GTPase-activating protein 45 OS=Homo sapiens GN=ARHGAP45 PE=1 SV=2 |
| RS5_HUMAN | 40S ribosomal protein S5 OS=Homo sapiens GN=RPS5 PE=1 SV=4 |
| RAB5C_HUMAN | Ras-related protein Rab-5C OS=Homo sapiens GN=RAB5C PE=1 SV=2 |
| ROA3_HUMAN | Heterogeneous nuclear ribonucleoprotein A3 OS=Homo sapiens GN=HNRNPA3 PE=1 SV=2 |
| PYGB_HUMAN | Glycogen phosphorylase, brain form OS=Homo sapiens GN=PYGB PE=1 SV=5 |
| LASP1_HUMAN | LIM and SH3 domain protein 1 OS=Homo sapiens GN=LASP1 PE=1 SV=2 |
| SRSF1_HUMAN | Serine/arginine-rich splicing factor 1 OS=Homo sapiens GN=SRSF1 PE=1 SV=2 |
| FABP5_HUMAN | Fatty acid-binding protein, epidermal OS=Homo sapiens GN=FABP5 PE=1 SV=3 |
| CBR1_HUMAN | Carbonyl reductase [NADPH] 1 OS=Homo sapiens GN=CBR1 PE=1 SV=3 |
| GNA13_HUMAN | Guanine nucleotide-binding protein subunit alpha-13 OS=Homo sapiens GN=GNA13 PE=1 SV=2 |
| HNRPU_HUMAN | Heterogeneous nuclear ribonucleoprotein U OS=Homo sapiens GN=HNRNPU PE=1 SV=6 |
| ILF2_HUMAN | Interleukin enhancer-binding factor 2 OS=Homo sapiens GN=ILF2 PE=1 SV=2 |
| PFKAL_HUMAN | ATP-dependent 6-phosphofructokinase, liver type OS=Homo sapiens GN=PFKL PE=1 SV=6 |
| PABP1_HUMAN | Polyadenylate-binding protein 1 OS=Homo sapiens GN=PABPC1 PE=1 SV=2 |
| PLCG2_HUMAN | 1-phosphatidylinositol 4,5-bisphosphate phosphodiesterase gamma-2 OS=Homo sapiens GN=PLCG2 PE=1 SV=4 |
| PSA2_HUMAN | Proteasome subunit alpha type-2 OS=Homo sapiens GN=PSMA2 PE=1 SV=2 |
| GPX1_HUMAN | Glutathione peroxidase 1 OS=Homo sapiens GN=GPX1 PE=1 SV=4 |
| DYN2_HUMAN | Dynamin-2 OS=Homo sapiens GN=DNM2 PE=1 SV=2 |
| GDIA_HUMAN | Rab GDP dissociation inhibitor alpha OS=Homo sapiens GN=GDI1 PE=1 SV=2 |
| SNP23_HUMAN | Synaptosomal-associated protein 23 OS=Homo sapiens GN=SNAP23 PE=1 SV=1 |
| ELAV1_HUMAN | ELAV-like protein 1 OS=Homo sapiens GN=ELAVL1 PE=1 SV=2 |
| SET_HUMAN | Protein SET OS=Homo sapiens GN=SET PE=1 SV=3 |
| PURA2_HUMAN | Adenylosuccinate synthetase isozyme 2 OS=Homo sapiens GN=ADSS PE=1 SV=3 |
| CD3E_HUMAN | T-cell surface glycoprotein CD3 epsilon chain OS=Homo sapiens GN=CD3E PE=1 SV=2 |
| CHM4B_HUMAN | Charged multivesicular body protein 4b OS=Homo sapiens GN=CHMP4B PE=1 SV=1 |
| AN32B_HUMAN | Acidic leucine-rich nuclear phosphoprotein 32 family member B OS=Homo sapiens GN=ANP32B PE=1 SV=1 |
| STXB3_HUMAN | Syntaxin-binding protein 3 OS=Homo sapiens GN=STXBP3 PE=1 SV=2 |
| STMN1_HUMAN | Stathmin OS=Homo sapiens GN=STMN1 PE=1 SV=3 |
| PRPS1_HUMAN | Ribose-phosphate pyrophosphokinase 1 OS=Homo sapiens GN=PRPS1 PE=1 SV=2 |
| SDHA_HUMAN | Succinate dehydrogenase [ubiquinone] flavoprotein subunit, mitochondrial OS=Homo sapiens GN=SDHA PE=1 SV=2 |
| PSB9_HUMAN | Proteasome subunit beta type-9 OS=Homo sapiens GN=PSMB9 PE=1 SV=2 |
| HPRT_HUMAN | Hypoxanthine-guanine phosphoribosyltransferase OS=Homo sapiens GN=HPRT1 PE=1 SV=2 |
| COX2_HUMAN | Cytochrome c oxidase subunit 2 OS=Homo sapiens GN=MT-CO2 PE=1 SV=1 |
| THAS_HUMAN | Thromboxane-A synthase OS=Homo sapiens GN=TBXAS1 PE=1 SV=3 |
| ALDH2_HUMAN | Aldehyde dehydrogenase, mitochondrial OS=Homo sapiens GN=ALDH2 PE=1 SV=2 |
| 41_HUMAN | Protein 4.1 OS=Homo sapiens GN=EPB41 PE=1 SV=4 |
| DHX9_HUMAN | ATP-dependent RNA helicase A OS=Homo sapiens GN=DHX9 PE=1 SV=4 |
| OTUB1_HUMAN | Ubiquitin thioesterase OTUB1 OS=Homo sapiens GN=OTUB1 PE=1 SV=2 |
| AMPB_HUMAN | Aminopeptidase B OS=Homo sapiens GN=RNPEP PE=1 SV=2 |
| WASP_HUMAN | Wiskott-Aldrich syndrome protein OS=Homo sapiens GN=WAS PE=1 SV=4 |
| TES_HUMAN | Testin OS=Homo sapiens GN=TES PE=1 SV=1 |
| ARP5L_HUMAN | Actin-related protein 2/3 complex subunit 5-like protein OS=Homo sapiens GN=ARPC5L PE=1 SV=1 |
| PNCB_HUMAN | Nicotinate phosphoribosyltransferase OS=Homo sapiens GN=NAPRT PE=1 SV=2 |
| SPB6_HUMAN | Serpin B6 OS=Homo sapiens GN=SERPINB6 PE=1 SV=3 |
| EIF3L_HUMAN | Eukaryotic translation initiation factor 3 subunit L OS=Homo sapiens GN=EIF3L PE=1 SV=1 |
| SYSC_HUMAN | Serine--tRNA ligase, cytoplasmic OS=Homo sapiens GN=SARS PE=1 SV=3 |
| KCD12_HUMAN | BTB/POZ domain-containing protein KCTD12 OS=Homo sapiens GN=KCTD12 PE=1 SV=1 |
| TRPV2_HUMAN | Transient receptor potential cation channel subfamily V member 2 OS=Homo sapiens GN=TRPV2 PE=1 SV=1 |
| CD81_HUMAN | CD81 antigen OS=Homo sapiens GN=CD81 PE=1 SV=1 |
| SIGL9_HUMAN | Sialic acid-binding Ig-like lectin 9 OS=Homo sapiens GN=SIGLEC9 PE=1 SV=2 |
| PEPD_HUMAN | Xaa-Pro dipeptidase OS=Homo sapiens GN=PEPD PE=1 SV=3 |
| SWP70_HUMAN | Switch-associated protein 70 OS=Homo sapiens GN=SWAP70 PE=1 SV=1 |
| AATM_HUMAN | Aspartate aminotransferase, mitochondrial OS=Homo sapiens GN=GOT2 PE=1 SV=3 |
| RALB_HUMAN | Ras-related protein Ral-B OS=Homo sapiens GN=RALB PE=1 SV=1 |
| BIN1_HUMAN | Myc box-dependent-interacting protein 1 OS=Homo sapiens GN=BIN1 PE=1 SV=1 |
| VNN2_HUMAN | Vascular non-inflammatory molecule 2 OS=Homo sapiens GN=VNN2 PE=1 SV=3 |
| SNX2_HUMAN | Sorting nexin-2 OS=Homo sapiens GN=SNX2 PE=1 SV=2 |
| IF2G_HUMAN | Eukaryotic translation initiation factor 2 subunit 3 OS=Homo sapiens GN=EIF2S3 PE=1 SV=3 |
| DPP3_HUMAN | Dipeptidyl peptidase 3 OS=Homo sapiens GN=DPP3 PE=1 SV=2 |
| DPP4_HUMAN | Dipeptidyl peptidase 4 OS=Homo sapiens GN=DPP4 PE=1 SV=2 |
| CLIC4_HUMAN | Chloride intracellular channel protein 4 OS=Homo sapiens GN=CLIC4 PE=1 SV=4 |
| NSF1C_HUMAN | NSFL1 cofactor p47 OS=Homo sapiens GN=NSFL1C PE=1 SV=2 |
| SYDC_HUMAN | Aspartate--tRNA ligase, cytoplasmic OS=Homo sapiens GN=DARS PE=1 SV=2 |
| GBB4_HUMAN | Guanine nucleotide-binding protein subunit beta-4 OS=Homo sapiens GN=GNB4 PE=1 SV=3 |
| SNAA_HUMAN | Alpha-soluble NSF attachment protein OS=Homo sapiens GN=NAPA PE=1 SV=3 |
| DHB4_HUMAN | Peroxisomal multifunctional enzyme type 2 OS=Homo sapiens GN=HSD17B4 PE=1 SV=3 |
| GLRX1_HUMAN | Glutaredoxin-1 OS=Homo sapiens GN=GLRX PE=1 SV=2 |
| PPIF_HUMAN | Peptidyl-prolyl cis-trans isomerase F, mitochondrial OS=Homo sapiens GN=PPIF PE=1 SV=1 |
| SYFB_HUMAN | Phenylalanine--tRNA ligase beta subunit OS=Homo sapiens GN=FARSB PE=1 SV=3 |
| DDX1_HUMAN | ATP-dependent RNA helicase DDX1 OS=Homo sapiens GN=DDX1 PE=1 SV=2 |
| SHPS1_HUMAN | Tyrosine-protein phosphatase non-receptor type substrate 1 OS=Homo sapiens GN=SIRPA PE=1 SV=2 |
| FYB_HUMAN | FYN-binding protein OS=Homo sapiens GN=FYB PE=1 SV=2 |
| RBMX_HUMAN | RNA-binding motif protein, X chromosome OS=Homo sapiens GN=RBMX PE=1 SV=3 |
| ABHEB_HUMAN | Protein ABHD14B OS=Homo sapiens GN=ABHD14B PE=1 SV=1 |
| ARF4_HUMAN | ADP-ribosylation factor 4 OS=Homo sapiens GN=ARF4 PE=1 SV=3 |
| GARS_HUMAN | Glycine--tRNA ligase OS=Homo sapiens GN=GARS PE=1 SV=3 |
| CD4_HUMAN | T-cell surface glycoprotein CD4 OS=Homo sapiens GN=CD4 PE=1 SV=1 |
| GBP1_HUMAN | Guanylate-binding protein 1 OS=Homo sapiens GN=GBP1 PE=1 SV=2 |
| MOT4_HUMAN | Monocarboxylate transporter 4 OS=Homo sapiens GN=SLC16A3 PE=1 SV=1 |
| FCGR1_HUMAN | High affinity immunoglobulin gamma Fc receptor I OS=Homo sapiens GN=FCGR1A PE=1 SV=2 |
| IMPA1_HUMAN | Inositol monophosphatase 1 OS=Homo sapiens GN=IMPA1 PE=1 SV=1 |
| HNRPL_HUMAN | Heterogeneous nuclear ribonucleoprotein L OS=Homo sapiens GN=HNRNPL PE=1 SV=2 |
| TSN_HUMAN | Translin OS=Homo sapiens GN=TSN PE=1 SV=1 |
| PP2BA_HUMAN | Serine/threonine-protein phosphatase 2B catalytic subunit alpha isoform OS=Homo sapiens GN=PPP3CA PE=1 SV=1 |
| SYRC_HUMAN | Arginine--tRNA ligase, cytoplasmic OS=Homo sapiens GN=RARS PE=1 SV=2 |
| VPS29_HUMAN | Vacuolar protein sorting-associated protein 29 OS=Homo sapiens GN=VPS29 PE=1 SV=1 |
| DCTN2_HUMAN | Dynactin subunit 2 OS=Homo sapiens GN=DCTN2 PE=1 SV=4 |
| SNX6_HUMAN | Sorting nexin-6 OS=Homo sapiens GN=SNX6 PE=1 SV=1 |
| PSMD3_HUMAN | 26S proteasome non-ATPase regulatory subunit 3 OS=Homo sapiens GN=PSMD3 PE=1 SV=2 |
| DCPS_HUMAN | m7GpppX diphosphatase OS=Homo sapiens GN=DCPS PE=1 SV=2 |
| SYYC_HUMAN | Tyrosine--tRNA ligase, cytoplasmic OS=Homo sapiens GN=YARS PE=1 SV=4 |
| GMPPB_HUMAN | Mannose-1-phosphate guanyltransferase beta OS=Homo sapiens GN=GMPPB PE=1 SV=2 |
| VATE1_HUMAN | V-type proton ATPase subunit E 1 OS=Homo sapiens GN=ATP6V1E1 PE=1 SV=1 |
| SC24C_HUMAN | Protein transport protein Sec24C OS=Homo sapiens GN=SEC24C PE=1 SV=3 |
| RAB32_HUMAN | Ras-related protein Rab-32 OS=Homo sapiens GN=RAB32 PE=1 SV=3 |
| AATC_HUMAN | Aspartate aminotransferase, cytoplasmic OS=Homo sapiens GN=GOT1 PE=1 SV=3 |
| APEX1_HUMAN | DNA-(apurinic or apyrimidinic site) lyase OS=Homo sapiens GN=APEX1 PE=1 SV=2 |
| EIF3D_HUMAN | Eukaryotic translation initiation factor 3 subunit D OS=Homo sapiens GN=EIF3D PE=1 SV=1 |
| CRLF3_HUMAN | Cytokine receptor-like factor 3 OS=Homo sapiens GN=CRLF3 PE=1 SV=2 |

Table S3. List of 16 proteins found exclusively in EVs derived from non-irradiated PBMCs.

| Accession | Protein Name |
| --- | --- |
| H2A1C_HUMAN | Histone H2A type 1-C OS=Homo sapiens GN=HIST1H2AC PE=1 SV=3 |
| H31_HUMAN | Histone H3.1 OS=Homo sapiens GN=HIST1H3A PE=1 SV=2 |
| SDCB1_HUMAN | Syntenin-1 OS=Homo sapiens GN=SDCBP PE=1 SV=1 |
| H13_HUMAN | Histone H1.3 OS=Homo sapiens GN=HIST1H1D PE=1 SV=2 |
| TTHY_HUMAN | Transthyretin OS=Homo sapiens GN=TTR PE=1 SV=1 |
| A1BG_HUMAN | Alpha-1B-glycoprotein OS=Homo sapiens GN=A1BG PE=1 SV=4 |
| HPT_HUMAN | Haptoglobin OS=Homo sapiens GN=HP PE=1 SV=1 |
| IGHA1_HUMAN | Immunoglobulin heavy constant alpha 1 OS=Homo sapiens GN=IGHA1 PE=1 SV=2 |
| LAMP1_HUMAN | Lysosome-associated membrane glycoprotein 1 OS=Homo sapiens GN=LAMP1 PE=1 SV=3 |
| IGSF8_HUMAN | Immunoglobulin superfamily member 8 OS=Homo sapiens GN=IGSF8 PE=1 SV=1 |
| MPRD_HUMAN | Cation-dependent mannose-6-phosphate receptor OS=Homo sapiens GN=M6PR PE=1 SV=1 |
| TRFE_HUMAN | Serotransferrin OS=Homo sapiens GN=TF PE=1 SV=3 |
| VPS4B_HUMAN | Vacuolar protein sorting-associated protein 4B OS=Homo sapiens GN=VPS4B PE=1 SV=2 |
| TFR1_HUMAN | Transferrin receptor protein 1 OS=Homo sapiens GN=TFRC PE=1 SV=2 |

**Table S4. List of 35 miRNAs identified in EVs derived from irradiated and non-irradiated PBMCs.**

| hsa-miR-24-3p |
| --- |
| hsa-miR-25-3p |
| hsa-miR-28-3p |
| hsa-miR-199a-3p |
| hsa-miR-30c-5p |
| hsa-miR-199b-3p |
| hsa-miR-204-3p |
| hsa-miR-214-3p |
| hsa-miR-223-3p |
| hsa-miR-30b-5p |
| hsa-miR-125b-5p |
| hsa-miR-126-3p |
| hsa-miR-151a-3p |
| hsa-miR-483-5p |
| hsa-miR-486-5p |
| hsa-miR-193b-3p |
| hsa-miR-500a-3p |
| hsa-miR-615-5p |
| hsa-miR-920 |
| hsa-miR-1246 |
| hsa-miR-320d |
| hsa-miR-320e |
| hsa-miR-3651 |
| hsa-miR-4429 |
| hsa-miR-4459 |
| hsa-miR-4487 |
| hsa-miR-4497 |
| hsa-miR-4688 |
| hsa-miR-5094 |
| hsa-miR-6126 |
| hsa-miR-6825-5p |
| hsa-miR-6780b-5p |
| hsa-miR-6870-5p |
| hsa-miR-7107-5p |
| hsa-miR-7111-5p |

**Table S5. List of 194 miRNAs identified only in EVs of irradiated PBMCs compared to EVs of non-irradiated PBMCs.**

| hsa-miR-21-5p |
| --- |
| hsa-miR-22-3p |
| hsa-miR-24-2-5p |
| hsa-miR-26a-1-3p |
| hsa-miR-28-5p |
| hsa-miR-29a-3p |
| hsa-miR-31-5p |
| hsa-miR-192-5p |
| hsa-miR-199a-5p |
| hsa-miR-30c-2-3p |
| hsa-miR-181a-2-3p |
| hsa-miR-182-5p |
| hsa-miR-222-3p |
| hsa-miR-27b-3p |
| hsa-miR-125b-1-3p |
| hsa-miR-128-3p |
| hsa-miR-140-5p |
| hsa-miR-152-3p |
| hsa-miR-125a-5p |
| hsa-miR-150-5p |
| hsa-miR-150-3p |
| hsa-miR-193a-5p |
| hsa-miR-194-5p |
| hsa-miR-200c-3p |
| hsa-miR-155-5p |
| hsa-miR-299-5p |
| hsa-miR-363-3p |
| hsa-miR-378a-5p |
| hsa-miR-383-3p |
| hsa-miR-342-5p |
| hsa-miR-326 |
| hsa-miR-148b-3p |
| hsa-miR-324-5p |
| hsa-miR-324-3p |
| hsa-miR-339-5p |
| hsa-miR-339-3p |
| hsa-miR-335-5p |
| hsa-miR-20b-3p |
| hsa-miR-146b-5p |
| hsa-miR-146b-3p |
| hsa-miR-512-3p |
| hsa-miR-519e-3p |
| hsa-miR-520f-5p |
| hsa-miR-502-3p |
| hsa-miR-504-3p |
| hsa-miR-505-5p |
| hsa-miR-92b-3p |
| hsa-miR-564 |
| hsa-miR-572 |
| hsa-miR-593-5p |
| hsa-miR-629-5p |
| hsa-miR-637 |
| hsa-miR-661 |
| hsa-miR-1301-3p |
| hsa-miR-885-3p |
| hsa-miR-543 |
| hsa-miR-939-5p |
| hsa-miR-1229-5p |
| hsa-miR-1231 |
| hsa-miR-1233-3p |
| hsa-miR-1238-5p |
| hsa-miR-1202 |
| hsa-miR-1290 |
| hsa-miR-548p |
| hsa-miR-548i |
| hsa-miR-1292-5p |
| hsa-miR-1324 |
| hsa-miR-1909-3p |
| hsa-miR-1910-5p |
| hsa-miR-2110 |
| hsa-miR-3130-3p |
| hsa-miR-3131 |
| hsa-miR-3146 |
| hsa-miR-3147 |
| hsa-miR-3154 |
| hsa-miR-3175 |
| hsa-miR-3180-3p |
| hsa-miR-3187-3p |
| hsa-miR-3197 |
| hsa-miR-3198 |
| hsa-miR-3199 |
| hsa-miR-4253 |
| hsa-miR-4266 |
| hsa-miR-3619-5p |
| hsa-miR-3679-5p |
| hsa-miR-3180 |
| hsa-miR-3937 |
| hsa-miR-378f |
| hsa-miR-378g |
| hsa-miR-4428 |
| hsa-miR-4430 |
| hsa-miR-4449 |
| hsa-miR-4451 |
| hsa-miR-4455 |
| hsa-miR-4462 |
| hsa-miR-4467 |
| hsa-miR-4469 |
| hsa-miR-4484 |
| hsa-miR-4419b |
| hsa-miR-2392 |
| hsa-miR-4513 |
| hsa-miR-4518 |
| hsa-miR-4525 |
| hsa-miR-4634 |
| hsa-miR-4640-5p |
| hsa-miR-4655-5p |
| hsa-miR-4665-3p |
| hsa-miR-4673 |
| hsa-miR-4676-5p |
| hsa-miR-4691-5p |
| hsa-miR-4708-3p |
| hsa-miR-4710 |
| hsa-miR-4714-5p |
| hsa-miR-4721 |
| hsa-miR-3064-5p |
| hsa-miR-371b-5p |
| hsa-miR-4784 |
| hsa-miR-4800-3p |
| hsa-miR-5006-5p |
| hsa-miR-5010-5p |
| hsa-miR-5572 |
| hsa-miR-548at-5p |
| hsa-miR-6080 |
| hsa-miR-6124 |
| hsa-miR-548az-3p |
| hsa-miR-6503-3p |
| hsa-miR-6511a-5p |
| hsa-miR-6716-5p |
| hsa-miR-6717-5p |
| hsa-miR-6721-5p |
| hsa-miR-6737-5p |
| hsa-miR-6738-5p |
| hsa-miR-6753-5p |
| hsa-miR-6754-3p |
| hsa-miR-6762-5p |
| hsa-miR-6763-5p |
| hsa-miR-6771-3p |
| hsa-miR-6774-5p |
| hsa-miR-6777-5p |
| hsa-miR-6782-5p |
| hsa-miR-6790-3p |
| hsa-miR-6797-5p |
| hsa-miR-6804-5p |
| hsa-miR-6805-3p |
| hsa-miR-6806-5p |
| hsa-miR-6808-5p |
| hsa-miR-6815-5p |
| hsa-miR-6817-5p |
| hsa-miR-6819-5p |
| hsa-miR-6829-5p |
| hsa-miR-6837-5p |
| hsa-miR-6840-3p |
| hsa-miR-6842-5p |
| hsa-miR-6877-5p |
| hsa-miR-6884-3p |
| hsa-miR-6887-5p |
| hsa-miR-6887-3p |
| hsa-miR-6893-5p |
| hsa-miR-7109-5p |
| hsa-miR-7114-5p |
| hsa-miR-7150 |
| hsa-miR-7161-3p |
| hsa-miR-7856-5p |
| hsa-miR-8064 |
| hsa-miR-8071 |
| hsa-miR-8073 |
| hsa-miR-8077 |
| hsa-mir-22 |
| hsa-mir-31 |
| hsa-mir-92a-1 |
| hsa-mir-302a |
| hsa-mir-409 |
| hsa-mir-484 |
| hsa-mir-500a |
| hsa-mir-611 |
| hsa-mir-628 |
| hsa-mir-631 |
| hsa-mir-635 |
| hsa-mir-1825 |
| hsa-mir-3117 |
| hsa-mir-500b |
| hsa-mir-3679 |
| hsa-mir-3926-1 |
| hsa-mir-3937 |
| hsa-mir-4449 |
| hsa-mir-3155b |
| hsa-mir-4516 |
| hsa-mir-4664 |
| hsa-mir-4707 |
| hsa-mir-4792 |
| hsa-mir-5189 |
| hsa-mir-6075 |
| hsa-mir-6088 |
| hsa-mir-6802 |

**Table S6. List of 188 miRNAs identified only in EVs derived from irradiated PBMCs.**

| hsa-miR-22-3p |
| --- |
| hsa-miR-24-3p |
| hsa-miR-24-2-5p |
| hsa-miR-25-3p |
| hsa-miR-26a-1-3p |
| hsa-miR-28-5p |
| hsa-miR-28-3p |
| hsa-miR-29a-3p |
| hsa-miR-192-5p |
| hsa-miR-199a-5p |
| hsa-miR-199a-3p |
| hsa-miR-30c-5p |
| hsa-miR-30c-2-3p |
| hsa-miR-181a-2-3p |
| hsa-miR-182-5p |
| hsa-miR-199b-3p |
| hsa-miR-204-3p |
| hsa-miR-214-3p |
| hsa-miR-222-3p |
| hsa-miR-223-3p |
| hsa-miR-27b-3p |
| hsa-miR-30b-5p |
| hsa-miR-125b-5p |
| hsa-miR-125b-1-3p |
| hsa-miR-128-3p |
| hsa-miR-152-3p |
| hsa-miR-126-3p |
| hsa-miR-150-5p |
| hsa-miR-150-3p |
| hsa-miR-193a-5p |
| hsa-miR-194-5p |
| hsa-miR-200c-3p |
| hsa-miR-155-5p |
| hsa-miR-299-5p |
| hsa-miR-363-3p |
| hsa-miR-378a-5p |
| hsa-miR-383-3p |
| hsa-miR-342-5p |
| hsa-miR-326 |
| hsa-miR-151a-3p |
| hsa-miR-148b-3p |
| hsa-miR-324-3p |
| hsa-miR-339-5p |
| hsa-miR-339-3p |
| hsa-miR-335-5p |
| hsa-miR-20b-3p |
| hsa-miR-483-5p |
| hsa-miR-486-5p |
| hsa-miR-146b-3p |
| hsa-miR-193b-3p |
| hsa-miR-512-3p |
| hsa-miR-519e-3p |
| hsa-miR-520f-5p |
| hsa-miR-500a-3p |
| hsa-miR-502-3p |
| hsa-miR-504-3p |
| hsa-miR-505-5p |
| hsa-miR-92b-3p |
| hsa-miR-564 |
| hsa-miR-593-5p |
| hsa-miR-615-5p |
| hsa-miR-629-5p |
| hsa-miR-885-3p |
| hsa-miR-543 |
| hsa-miR-920 |
| hsa-miR-1229-5p |
| hsa-miR-1231 |
| hsa-miR-1233-3p |
| hsa-miR-1238-5p |
| hsa-miR-1290 |
| hsa-miR-1246 |
| hsa-miR-548p |
| hsa-miR-548i |
| hsa-miR-1292-5p |
| hsa-miR-1324 |
| hsa-miR-320d |
| hsa-miR-1909-3p |
| hsa-miR-2110 |
| hsa-miR-3130-3p |
| hsa-miR-3131 |
| hsa-miR-3146 |
| hsa-miR-3154 |
| hsa-miR-3180-3p |
| hsa-miR-320e |
| hsa-miR-3197 |
| hsa-miR-3199 |
| hsa-miR-4253 |
| hsa-miR-4266 |
| hsa-miR-3619-5p |
| hsa-miR-3651 |
| hsa-miR-3180 |
| hsa-miR-3937 |
| hsa-miR-378f |
| hsa-miR-378g |
| hsa-miR-4428 |
| hsa-miR-4429 |
| hsa-miR-4430 |
| hsa-miR-4449 |
| hsa-miR-4455 |
| hsa-miR-4459 |
| hsa-miR-4467 |
| hsa-miR-4469 |
| hsa-miR-4484 |
| hsa-miR-4487 |
| hsa-miR-4497 |
| hsa-miR-4419b |
| hsa-miR-2392 |
| hsa-miR-4513 |
| hsa-miR-4518 |
| hsa-miR-4525 |
| hsa-miR-4640-5p |
| hsa-miR-4655-5p |
| hsa-miR-4665-3p |
| hsa-miR-4676-5p |
| hsa-miR-4688 |
| hsa-miR-4691-5p |
| hsa-miR-4708-3p |
| hsa-miR-4714-5p |
| hsa-miR-4721 |
| hsa-miR-3064-5p |
| hsa-miR-4784 |
| hsa-miR-4800-3p |
| hsa-miR-5010-5p |
| hsa-miR-5094 |
| hsa-miR-6080 |
| hsa-miR-6126 |
| hsa-miR-548az-3p |
| hsa-miR-6511a-5p |
| hsa-miR-6716-5p |
| hsa-miR-6717-5p |
| hsa-miR-6721-5p |
| hsa-miR-6737-5p |
| hsa-miR-6738-5p |
| hsa-miR-6754-3p |
| hsa-miR-6762-5p |
| hsa-miR-6763-5p |
| hsa-miR-6771-3p |
| hsa-miR-6774-5p |
| hsa-miR-6777-5p |
| hsa-miR-6782-5p |
| hsa-miR-6790-3p |
| hsa-miR-6804-5p |
| hsa-miR-6805-3p |
| hsa-miR-6808-5p |
| hsa-miR-6815-5p |
| hsa-miR-6817-5p |
| hsa-miR-6825-5p |
| hsa-miR-6829-5p |
| hsa-miR-6780b-5p |
| hsa-miR-6842-5p |
| hsa-miR-6870-5p |
| hsa-miR-6877-5p |
| hsa-miR-6887-5p |
| hsa-miR-6887-3p |
| hsa-miR-6893-5p |
| hsa-miR-7107-5p |
| hsa-miR-7109-5p |
| hsa-miR-7111-5p |
| hsa-miR-7150 |
| hsa-miR-7161-3p |
| hsa-miR-7856-5p |
| hsa-miR-8071 |
| hsa-miR-8077 |
| hsa-mir-22 |
| hsa-mir-31 |
| hsa-mir-92a-1 |
| hsa-mir-302a |
| hsa-mir-409 |
| hsa-mir-484 |
| hsa-mir-500a |
| hsa-mir-611 |
| hsa-mir-628 |
| hsa-mir-631 |
| hsa-mir-635 |
| hsa-mir-1825 |
| hsa-mir-3117 |
| hsa-mir-3679 |
| hsa-mir-3926-1 |
| hsa-mir-4449 |
| hsa-mir-3155b |
| hsa-mir-4516 |
| hsa-mir-4664 |
| hsa-mir-4707 |
| hsa-mir-4792 |
| hsa-mir-5189 |
| hsa-mir-6075 |
| hsa-mir-6088 |
| hsa-mir-6802 |

**Table S7. List of 455 miRNAs identified only in irradiated PBMCs.**

| hsa-miR-19a-3p |
| --- |
| hsa-miR-33a-5p |
| hsa-miR-29b-3p |
| hsa-miR-197-5p |
| hsa-miR-129-5p |
| hsa-miR-34a-5p |
| hsa-miR-181c-5p |
| hsa-miR-215-3p |
| hsa-miR-216a-5p |
| hsa-miR-129-2-3p |
| hsa-miR-186-5p |
| hsa-miR-188-5p |
| hsa-miR-194-3p |
| hsa-miR-106b-5p |
| hsa-miR-30c-1-3p |
| hsa-miR-99b-5p |
| hsa-miR-130b-5p |
| hsa-miR-365a-5p |
| hsa-miR-373-5p |
| hsa-miR-383-5p |
| hsa-miR-424-5p |
| hsa-miR-431-3p |
| hsa-miR-518c-3p |
| hsa-miR-519a-3p |
| hsa-miR-455-3p |
| hsa-miR-555 |
| hsa-miR-574-5p |
| hsa-miR-550a-5p |
| hsa-miR-595 |
| hsa-miR-598-5p |
| hsa-miR-614 |
| hsa-miR-616-5p |
| hsa-miR-617 |
| hsa-miR-618 |
| hsa-miR-619-5p |
| hsa-miR-621 |
| hsa-miR-622 |
| hsa-miR-623 |
| hsa-miR-628-3p |
| hsa-miR-634 |
| hsa-miR-638 |
| hsa-miR-639 |
| hsa-miR-646 |
| hsa-miR-648 |
| hsa-miR-652-5p |
| hsa-miR-659-3p |
| hsa-miR-671-5p |
| hsa-miR-671-3p |
| hsa-miR-668-5p |
| hsa-miR-1224-5p |
| hsa-miR-762 |
| hsa-miR-298 |
| hsa-miR-665 |
| hsa-miR-760 |
| hsa-miR-301b |
| hsa-miR-937-5p |
| hsa-miR-1180-3p |
| hsa-miR-1181 |
| hsa-miR-1183 |
| hsa-miR-1227-5p |
| hsa-miR-1233-5p |
| hsa-miR-1236-3p |
| hsa-miR-1207-5p |
| hsa-miR-1208 |
| hsa-miR-1261 |
| hsa-miR-1268a |
| hsa-miR-1281 |
| hsa-miR-1292-3p |
| hsa-miR-664a-5p |
| hsa-miR-1306-5p |
| hsa-miR-1306-3p |
| hsa-miR-1538 |
| hsa-miR-1825 |
| hsa-miR-1915-3p |
| hsa-miR-1972 |
| hsa-miR-2861 |
| hsa-miR-3129-3p |
| hsa-miR-3148 |
| hsa-miR-3150a-3p |
| hsa-miR-3151-3p |
| hsa-miR-3074-3p |
| hsa-miR-3157-3p |
| hsa-miR-3162-5p |
| hsa-miR-3181 |
| hsa-miR-3185 |
| hsa-miR-3186-3p |
| hsa-miR-3187-5p |
| hsa-miR-3188 |
| hsa-miR-3189-3p |
| hsa-miR-3192-5p |
| hsa-miR-3200-5p |
| hsa-miR-3201 |
| hsa-miR-4296 |
| hsa-miR-4301 |
| hsa-miR-4299 |
| hsa-miR-4298 |
| hsa-miR-4260 |
| hsa-miR-4270 |
| hsa-miR-4281 |
| hsa-miR-4284 |
| hsa-miR-4290 |
| hsa-miR-3605-5p |
| hsa-miR-3609 |
| hsa-miR-3615 |
| hsa-miR-3616-3p |
| hsa-miR-3620-5p |
| hsa-miR-3621 |
| hsa-miR-3659 |
| hsa-miR-3663-3p |
| hsa-miR-3665 |
| hsa-miR-3668 |
| hsa-miR-3687 |
| hsa-miR-3692-3p |
| hsa-miR-3907 |
| hsa-miR-3916 |
| hsa-miR-3921 |
| hsa-miR-3922-5p |
| hsa-miR-3936 |
| hsa-miR-3940-5p |
| hsa-miR-1268b |
| hsa-miR-4417 |
| hsa-miR-4433-3p |
| hsa-miR-4440 |
| hsa-miR-4463 |
| hsa-miR-548aj-3p |
| hsa-miR-4474-3p |
| hsa-miR-4481 |
| hsa-miR-4482-3p |
| hsa-miR-4485 |
| hsa-miR-4486 |
| hsa-miR-4496 |
| hsa-miR-4502 |
| hsa-miR-4505 |
| hsa-miR-4507 |
| hsa-miR-4508 |
| hsa-miR-4516 |
| hsa-miR-4526 |
| hsa-miR-4530 |
| hsa-miR-4533 |
| hsa-miR-548am-3p |
| hsa-miR-1587 |
| hsa-miR-4539 |
| hsa-miR-4632-5p |
| hsa-miR-4644 |
| hsa-miR-4647 |
| hsa-miR-4651 |
| hsa-miR-4653-3p |
| hsa-miR-4657 |
| hsa-miR-4669 |
| hsa-miR-4670-3p |
| hsa-miR-4674 |
| hsa-miR-4684-3p |
| hsa-miR-4685-5p |
| hsa-miR-4687-3p |
| hsa-miR-4689 |
| hsa-miR-4691-3p |
| hsa-miR-4695-5p |
| hsa-miR-4700-5p |
| hsa-miR-4701-3p |
| hsa-miR-4707-5p |
| hsa-miR-4713-3p |
| hsa-miR-4717-3p |
| hsa-miR-4722-5p |
| hsa-miR-4730 |
| hsa-miR-4731-3p |
| hsa-miR-4734 |
| hsa-miR-4741 |
| hsa-miR-4745-5p |
| hsa-miR-4745-3p |
| hsa-miR-4746-3p |
| hsa-miR-4758-5p |
| hsa-miR-4763-3p |
| hsa-miR-4767 |
| hsa-miR-4772-5p |
| hsa-miR-4436b-5p |
| hsa-miR-4788 |
| hsa-miR-4790-3p |
| hsa-miR-4793-3p |
| hsa-miR-5001-5p |
| hsa-miR-5093 |
| hsa-miR-1273f |
| hsa-miR-5187-3p |
| hsa-miR-5189-5p |
| hsa-miR-5195-5p |
| hsa-miR-5195-3p |
| hsa-miR-664b-5p |
| hsa-miR-548at-3p |
| hsa-miR-5585-3p |
| hsa-miR-5588-3p |
| hsa-miR-5591-3p |
| hsa-miR-1199-5p |
| hsa-miR-6068 |
| hsa-miR-6075 |
| hsa-miR-6084 |
| hsa-miR-6088 |
| hsa-miR-6089 |
| hsa-miR-6127 |
| hsa-miR-6132 |
| hsa-miR-6133 |
| hsa-miR-6500-3p |
| hsa-miR-6722-3p |
| hsa-miR-6723-5p |
| hsa-miR-6724-5p |
| hsa-miR-6727-5p |
| hsa-miR-6731-5p |
| hsa-miR-6736-5p |
| hsa-miR-6737-3p |
| hsa-miR-6744-3p |
| hsa-miR-6746-5p |
| hsa-miR-6747-5p |
| hsa-miR-6749-5p |
| hsa-miR-6751-5p |
| hsa-miR-6752-5p |
| hsa-miR-6756-5p |
| hsa-miR-6757-5p |
| hsa-miR-6771-5p |
| hsa-miR-6775-5p |
| hsa-miR-6779-5p |
| hsa-miR-6784-5p |
| hsa-miR-6785-5p |
| hsa-miR-6786-5p |
| hsa-miR-6786-3p |
| hsa-miR-6787-3p |
| hsa-miR-6789-5p |
| hsa-miR-6790-5p |
| hsa-miR-6791-5p |
| hsa-miR-6792-5p |
| hsa-miR-6793-5p |
| hsa-miR-6799-5p |
| hsa-miR-6800-5p |
| hsa-miR-6800-3p |
| hsa-miR-6803-5p |
| hsa-miR-6812-5p |
| hsa-miR-6813-5p |
| hsa-miR-6820-5p |
| hsa-miR-6821-5p |
| hsa-miR-6827-5p |
| hsa-miR-6831-5p |
| hsa-miR-6836-5p |
| hsa-miR-6838-5p |
| hsa-miR-6847-5p |
| hsa-miR-6849-5p |
| hsa-miR-6850-5p |
| hsa-miR-6850-3p |
| hsa-miR-6851-3p |
| hsa-miR-6854-5p |
| hsa-miR-6856-5p |
| hsa-miR-6857-3p |
| hsa-miR-6858-5p |
| hsa-miR-6871-5p |
| hsa-miR-6873-3p |
| hsa-miR-6877-3p |
| hsa-miR-6878-3p |
| hsa-miR-6879-3p |
| hsa-miR-6883-5p |
| hsa-miR-6889-3p |
| hsa-miR-6891-3p |
| hsa-miR-7106-3p |
| hsa-miR-7108-5p |
| hsa-miR-7112-3p |
| hsa-miR-7113-3p |
| hsa-miR-7152-3p |
| hsa-miR-7155-5p |
| hsa-miR-7156-5p |
| hsa-miR-7160-5p |
| hsa-miR-7162-3p |
| hsa-miR-4433b-3p |
| hsa-miR-7844-5p |
| hsa-miR-7845-5p |
| hsa-miR-7847-3p |
| hsa-miR-7851-3p |
| hsa-miR-8052 |
| hsa-miR-8055 |
| hsa-miR-8060 |
| hsa-miR-8063 |
| hsa-mir-29a |
| hsa-mir-197 |
| hsa-mir-30c-2 |
| hsa-mir-210 |
| hsa-mir-23b |
| hsa-mir-152 |
| hsa-mir-125a |
| hsa-mir-206 |
| hsa-mir-30e |
| hsa-mir-326 |
| hsa-mir-339 |
| hsa-mir-520a |
| hsa-mir-516b-1 |
| hsa-mir-502 |
| hsa-mir-502 |
| hsa-mir-509-1 |
| hsa-mir-572 |
| hsa-mir-548a-3 |
| hsa-mir-610 |
| hsa-mir-622 |
| hsa-mir-623 |
| hsa-mir-638 |
| hsa-mir-639 |
| hsa-mir-548d-1 |
| hsa-mir-663a |
| hsa-mir-449b |
| hsa-mir-766 |
| hsa-mir-509-2 |
| hsa-mir-876 |
| hsa-mir-885 |
| hsa-mir-509-3 |
| hsa-mir-940 |
| hsa-mir-1227 |
| hsa-mir-1228 |
| hsa-mir-1234 |
| hsa-mir-1236 |
| hsa-mir-1202 |
| hsa-mir-1205 |
| hsa-mir-1305 |
| hsa-mir-548f-5 |
| hsa-mir-548f-5 |
| hsa-mir-1248 |
| hsa-mir-1254-1 |
| hsa-mir-1262 |
| hsa-mir-1265 |
| hsa-mir-548h-3 |
| hsa-mir-548h-3 |
| hsa-mir-548h-4 |
| hsa-mir-664a |
| hsa-mir-1306 |
| hsa-mir-1909 |
| hsa-mir-1910 |
| hsa-mir-1913 |
| hsa-mir-1914 |
| hsa-mir-3127 |
| hsa-mir-466 |
| hsa-mir-3141 |
| hsa-mir-3158-1 |
| hsa-mir-3160-1 |
| hsa-mir-3161 |
| hsa-mir-3185 |
| hsa-mir-3191 |
| hsa-mir-3202-1 |
| hsa-mir-3202-1 |
| hsa-mir-4310 |
| hsa-mir-4317 |
| hsa-mir-4258 |
| hsa-mir-4326 |
| hsa-mir-4270 |
| hsa-mir-4275 |
| hsa-mir-4281 |
| hsa-mir-4329 |
| hsa-mir-500b |
| hsa-mir-3619 |
| hsa-mir-3648 |
| hsa-mir-3661 |
| hsa-mir-3687 |
| hsa-mir-3689a |
| hsa-mir-3689b |
| hsa-mir-3689b |
| hsa-mir-3909 |
| hsa-mir-3150b |
| hsa-mir-3921 |
| hsa-mir-548ab |
| hsa-mir-548ab |
| hsa-mir-4417 |
| hsa-mir-4436a |
| hsa-mir-548ae-2 |
| hsa-mir-4442 |
| hsa-mir-4444-1 |
| hsa-mir-4446 |
| hsa-mir-548ag-1 |
| hsa-mir-4459 |
| hsa-mir-548aj-1 |
| hsa-mir-4479 |
| hsa-mir-4495 |
| hsa-mir-4497 |
| hsa-mir-4508 |
| hsa-mir-4515 |
| hsa-mir-4522 |
| hsa-mir-4523 |
| hsa-mir-4538 |
| hsa-mir-4634 |
| hsa-mir-4644 |
| hsa-mir-4658 |
| hsa-mir-4679-1 |
| hsa-mir-4679-2 |
| hsa-mir-4683 |
| hsa-mir-1343 |
| hsa-mir-4690 |
| hsa-mir-4694 |
| hsa-mir-4695 |
| hsa-mir-4697 |
| hsa-mir-4698 |
| hsa-mir-4701 |
| hsa-mir-4705 |
| hsa-mir-4714 |
| hsa-mir-4717 |
| hsa-mir-4719 |
| hsa-mir-4735 |
| hsa-mir-4740 |
| hsa-mir-4741 |
| hsa-mir-4766 |
| hsa-mir-4779 |
| hsa-mir-3688-2 |
| hsa-mir-4800 |
| hsa-mir-5010 |
| hsa-mir-5095 |
| hsa-mir-5192 |
| hsa-mir-5194 |
| hsa-mir-5195 |
| hsa-mir-4444-2 |
| hsa-mir-5586 |
| hsa-mir-4536-2 |
| hsa-mir-5590 |
| hsa-mir-5682 |
| hsa-mir-5703 |
| hsa-mir-6072 |
| hsa-mir-6089-1 |
| hsa-mir-6090 |
| hsa-mir-6503 |
| hsa-mir-6511a-1 |
| hsa-mir-6715a |
| hsa-mir-6511b-1 |
| hsa-mir-6720 |
| hsa-mir-6729 |
| hsa-mir-6736 |
| hsa-mir-6766 |
| hsa-mir-6768 |
| hsa-mir-6775 |
| hsa-mir-6779 |
| hsa-mir-6789 |
| hsa-mir-6790 |
| hsa-mir-6796 |
| hsa-mir-6800 |
| hsa-mir-6800 |
| hsa-mir-6815 |
| hsa-mir-6836 |
| hsa-mir-6842 |
| hsa-mir-6850 |
| hsa-mir-6858 |
| hsa-mir-6769b |
| hsa-mir-6860 |
| hsa-mir-6863 |
| hsa-mir-6866 |
| hsa-mir-6869 |
| hsa-mir-6890 |
| hsa-mir-7114 |
| hsa-mir-6511b-2 |
| hsa-mir-6089-2 |
| hsa-mir-6511a-2 |
| hsa-mir-6511a-3 |
| hsa-mir-6511a-4 |
| hsa-mir-7159 |
| hsa-mir-486-2 |
| hsa-mir-7162 |
| hsa-mir-8070 |
| hsa-mir-8072 |
| hsa-mir-8088 |
| hsa-mir-8089 |

**Table S8. List of 41 miRNAs identified in PBMCs and EVs after irradiation.**

| hsa-miR-21-5p |
| --- |
| hsa-miR-31-5p |
| hsa-miR-140-5p |
| hsa-miR-125a-5p |
| hsa-miR-324-5p |
| hsa-miR-146b-5p |
| hsa-miR-572 |
| hsa-miR-637 |
| hsa-miR-661 |
| hsa-miR-1301-3p |
| hsa-miR-939-5p |
| hsa-miR-1202 |
| hsa-miR-1910-5p |
| hsa-miR-3147 |
| hsa-miR-3175 |
| hsa-miR-3187-3p |
| hsa-miR-3198 |
| hsa-miR-3679-5p |
| hsa-miR-4451 |
| hsa-miR-4462 |
| hsa-miR-4634 |
| hsa-miR-4673 |
| hsa-miR-4710 |
| hsa-miR-371b-5p |
| hsa-miR-5006-5p |
| hsa-miR-5572 |
| hsa-miR-548at-5p |
| hsa-miR-6124 |
| hsa-miR-6503-3p |
| hsa-miR-6753-5p |
| hsa-miR-6797-5p |
| hsa-miR-6806-5p |
| hsa-miR-6819-5p |
| hsa-miR-6837-5p |
| hsa-miR-6840-3p |
| hsa-miR-6884-3p |
| hsa-miR-7114-5p |
| hsa-miR-8064 |
| hsa-miR-8073 |
| hsa-mir-500b |
| hsa-mir-3937 |

**Table S9. Individual donor information.**

| **demographic data** | acceptance criteria | unit | **donor 1** | **donor 2** | **donor 3** | **donor 4** | **donor 5** | **donor 6** | **donor 7** | **donor 8** | **donor 9** | **donor 10** | **SD** |
| --- | --- | --- | --- | --- | --- | --- | --- | --- | --- | --- | --- | --- | --- |
| age | 18-65 | years | 38 | 46 | 54 | 39 | 32 | 55 | 20 | 59 | 33 | 24 | 12.6 |
| sex | m | n.a. | m | m | m | m | m | m | m | m | m | m |  |
| body weight | > 50 kg | kg | 93 | 90 | 79 | 76 | 67 | 95 | 75 | 70 | 75 | 107 | 12.2 |
| body height | n.a. | cm | 173 | 181 | 172 | 177 | 179 | 185 | 175 | 162 | 184 | 177 | 6.3 |
| BMI | t.b.d. | kg/m^2^ | 31.07 | 27.47 | 26.70 | 24.26 | 20.91 | 27.76 | 24.49 | 26.67 | 22.15 | 34.15 | 3.8 |
| **cell count & viability** |  |  |  |  |  |  |  |  |  |  |  |  |  |
| leukocyte count from whole blood | ≤ 16 | x 10^3^/µl | 8.44 | 11.25 | 7.79 | 5.86 | 8.8 | 10.98 | 8.36 | 7.67 | 7.42 | 11.2 | 1.7 |
| apoptotic cells | n.a. | % | 14.29 | 19.62 | 19.8 | 21.57 | 15.63 | 20.02 | 16.05 | 18.78 | 19.13 | 18 | 2.2 |
| necrotic cells | n.a. | % | 6.29 | 7.51 | 6.15 | 5.53 | 4.19 | 5.18 | 6.03 | 3.83 | 5.85 | 5.73 | 1.01 |
| vital cells | > 50% | % | 79.42 | 72.87 | 74.05 | 72.9 | 80.18 | 74.8 | 77.92 | 77.39 | 75.02 | 76.27 | 2.5 |

**Table S10. Averaged donor data of 10 pooled MNC^aposec^.**

| **averaged demographic data** | unit | **pool 1** | **pool 2** | **pool 3** | **pool 4** | **pool 5** | **pool 6** | **pool 7** | **pool 8** | **pool 9** | **pool 10** | **SD** |
| --- | --- | --- | --- | --- | --- | --- | --- | --- | --- | --- | --- | --- |
| age | years | 34.3 | 38.7 | 42.2 | 36.4 | 45.2 | 37 | 29.8 | 30.5 | 41.5 | 48.3 | 5.7 |
| body weight | kg | 90.4 | 75.2 | 83.1 | 78.9 | 81.6 | 74.4 | 87.2 | 86.8 | 84.2 | 85.3 | 4.9 |
| body height | cm | 182.6 | 177.6 | 176.3 | 176.6 | 178.9 | 174.2 | 180 | 178.9 | 177.7 | 179.4 | 2.2 |
| BMI | kg/m2 | 27.02 | 23.78 | 26.73 | 25.46 | 25.41 | 24.44 | 26.92 | 27.17 | 26.64 | 26.43 | 1.1 |
| **averaged cell counts & viabilities** | |  |  |  |  |  |  |  |  |  |  |  |
| leukocyte count from whole blood | x 10^3^/µl | 8.31 | 7.39 | 7.67 | 8.86 | 7.18 | 7.16 | 8.31 | 8.83 | 8.74 | 8.09 | 0.63 |
| apoptotic cells | % | 25.21 | 31.11 | 29.61 | 26.05 | 27.03 | 28.34 | 29.16 | 35.27 | 29.63 | 26.91 | 2.8 |
| necrotic cells | % | 6.98 | 6.42 | 1.26 | 0.77 | 1.4 | 1.26 | 1.24 | 3.51 | 1.28 | 1.69 | 2.2 |
| vital cells | % | 67.82 | 62.47 | 69.13 | 73.18 | 71.6 | 70.4 | 69.604 | 61.22 | 69.09 | 71.39 | 3.7 |

**Table S11. Variability of TGF-β1 and IL-8 between individual donors and pooled MNC^aposec^.**

| TGF-β1 (pg/mL) | **donor 1** | **donor 2** | **donor 3** | **donor 4** | **SD** |
| --- | --- | --- | --- | --- | --- |
|  | 3554.8 | 3722.03 | 8261.7 | 6009.7 | **1922.3** |
|  | **pool 1** | **pool 2** | **pool 3** | **pool 4** |  |
|  | 5286.6 | 3881.9 | 4222.1 | 3413.3 | **689.4** |
| IL-8 (pg/mL) | **donor 1** | **donor 2** | **donor 3** | **donor 4** |  |
|  | 1119.6 | 1548.4 | 1475.7 | 1196.9 | **180.8** |
|  | **pool 1** | **pool 2** | **pool 3** | **pool 4** |  |
|  | 1368.8 | 1358.8 | 1414.4 | 1256.2 | **57.8** |
